# Hyperbaric oxygen therapy compared to pharmacological intervention in fibromyalgia patients following traumatic brain injury: A randomized, controlled trial

**DOI:** 10.1371/journal.pone.0282406

**Published:** 2023-03-10

**Authors:** Jacob N. Ablin, Erez Lang, Merav Catalogna, Valerie Aloush, Amir Hadanny, Keren Doenyas-Barak, Shachar Finci, Nir Polak, Gregory Fishlev, Calanit Korin, Rachel Yehudit Tzidky, Oshra Meir Genuth, Shai Efrati

**Affiliations:** 1 Tel Aviv Sourasky Medical Center, Tel-Aviv, Israel; 2 Sackler School of Medicine, Tel- Aviv University, Tel-Aviv, Israel; 3 Sagol Center for Hyperbaric Medicine and Research, Shamir (Assaf Harofeh) Medical Center, Be’er Ya’akov, Israel; 4 Sagol School of Neuroscience, Tel-Aviv University, Tel-Aviv, Israel; Alanya Alaaddin Keykubat University: Alanya Alaaddin Keykubat Universitesi, TURKEY

## Abstract

Fibromyalgia is a chronic pain syndrome with unsatisfactory response to current treatments. Physical trauma, including traumatic brain Injury (TBI) is among the etiological triggers. Hyperbaric Oxygen therapy (HBOT) is an intervention that combines 100% oxygen with elevated atmospheric pressure. HBOT has been applied as a neuro-modulatory treatment in central nervous system–related conditions. The current study investigated the utility of HBOT for TBI–related fibromyalgia. Fibromyalgia patients with a history of TBI were randomized to either HBOT or pharmacological intervention. HBOT protocol comprised 60 daily sessions, breathing 100% oxygen by mask at 2 absolute atmospheres (ATA) for 90 minutes. Pharmacological treatment included Pregabalin or Duloxetine. The primary outcome was subjective pain intensity on visual analogue scale (VAS); Secondary endpoints included questionnaires assessing fibromyalgia symptoms as well as Tc-99m-ECD SPECT brain imaging. Pain threshold and conditioned pain modulation (CPM) were also assessed. Results demonstrated a significant group-by-time interaction in pain intensity post-HBOT compared to the medication group (p = 0.001), with a large net effect size (d = -0.95) in pain intensity reduction following HBOT compared to medications. Fibromyalgia related symptoms and pain questionnaires demonstrated significant improvements induced by HBOT as well as improvements in quality of life and increase in pain thresholds and CPM. SPECT demonstrated significant group-by-time interactions between HBOT and medication groups in the left frontal and the right temporal cortex. In conclusion, HBOT can improve pain symptoms, quality of life, emotional and social function of patients suffering from FMS triggered by TBI. The beneficial clinical effect is correlated with increased brain activity in frontal and parietal regions, associated with executive function and emotional processing.

## Introduction

Fibromyalgia Syndrome (FMS) is a common, chronic condition clinically characterized by widespread pain, dyscognition and associated symptoms [1]. FMS is currently considered to be a prototype of nociplastic pain–conditions, in which pain is attributed to aberrant processing within the central nervous system, rather than to peripheral damage to tissue or nerves [2, 3]. Fibromyalgia affects 2–8% of the general population and has been estimated to generate 3–5 fold greater health care costs than the healthy population [4].

Current treatment for FMS relies on multidisciplinary interventions, with only a modest role for pharmacological agents [5, 6]. Notably, even with optimal implementation of such methods, which are not universally accessible, success rates remain modest, and FMS continues to constitute an unmet need.

While the etiology of FMS remains incompletely understood, the interaction between genetic predisposition and exposure to various types of triggers is considered to underlie many cases. Among these triggers, traumatic brain injury (TBI), resulting from an external mechanical force, such as an impact during a fall, a rapid acceleration or deceleration during a motor vehicle accident or a blast wave, has frequently been described as an inciting factor [7–9]. Patients suffering from TBI-induced FMS may be considered a relatively distinct subgroup, with a clearly defined mechanism of injury and time of initiation. Notably, previous research has usually not focused on studying this subgroup of FMS patients separately, but rather has tended to scrutinize heterogenetic patient populations.

Hyperbaric oxygen therapy (HBOT) is a medical treatment in which 100% oxygen is administered at an environmental pressure greater than one atmosphere absolute (ATA) [10]. While currently FDA–approved indications for HBOT are limited (e.g., decompression sickness, non-healing wounds etc.), certain HBOT protocols are being used to induce neuroplasticity in several types of brain injuries, including traumatic brain injury (TBI), post- stroke, post-traumatic stress disorder, age related cognitive decline, among other neurological syndromes [11–20]. Previous studies, performed both by our group and by others, have demonstrated the efficacy of HBOT as a treatment modality for FMS patients [21, 22]. HBOT was shown to induce significant clinical improvement, associated with documentable neuroplasticity, evaluated by brain imaging [23, 24]. Furthermore, HBOT was shown to be a promising modality for the subgroup of FMS patients who developed the syndrome with a history of severe emotional stress such as childhood abuse [24, 25].

In the current study, we have attempted to evaluate the therapeutic effect of HBOT on FMS patients with a clear clinical history of TBI and to compare it to the current standard pharmacological treatment. Our objective was to ascertain the clinical utility of this treatment in this specific subgroup of FMS patients, as well as studying the neuroplasticity effects of the intervention through metabolic brain imaging.

## Methods

### Patients

Study inclusion criteria included age ≥18 years old, diagnosis of FMS according to the updated 2016 diagnostic criteria [26], and with a history of TBI. Patients were excluded if they currently or previously were treated with Duloxetine or Pregabalin, were diagnosed with systemic inflammatory disorders including rheumatological and autoimmune disease, with chronic ongoing infection, with major psychiatric disorders (excluding anxiety), previous neurologic conditions (Epilepsy, neuromuscular diseases, metabolic diseases, etc.) active malignancy, previous treatment with HBOT for any reason, chest pathology, inner ear disease, and inability to provide informed consent.

### Trial design

A prospective randomized, single blind (outcomes assessors), controlled trial was conducted at the Shamir Medical Center (SMC), Israel. After signing an informed consent, patients were randomized to either HBOT or pharmacological intervention groups in a 1:1 ratio according to a computerized randomization table, supervised by a blinded researcher. Evaluation was performed at baseline for both groups and 1–3 weeks after the last HBOT session or three months of medications protocol. All evaluators were blinded to the patients’ group allocation. Intention to treat analysis was performed on all included patients. The study was approved by Shamir Medical Center’s institutional review board (IRB) (No. 058-17-ASF) and all participants signed an informed consent prior to their inclusion. All research was performed according to the relevant guidelines and regulations. This study was registered with ClinicalTrials.gov, number NCT03325959 on 30/10/2017.

### Intervention

HBOT protocol was administrated in a multi-place Starmed-2700 chamber (HAUX, Germany). The protocol comprised 60 daily sessions, five sessions per week, within a three-month period. The HBOT protocol included breathing 100% oxygen by mask at 2ATA for 90 minutes with five-minute air breaks every 20 minutes. Compression/decompression rates were 1.0 meter/minute.

Patients in the medication group were assigned to receive pharmacological intervention for three-month period, with one of the two medications currently approved in Israel for FMS, Duloxetine and Pregabalin, as outlined in the local rheumatology guidelines for the diagnosis and treatment of FMS [27]. Drugs were chosen by the treating rheumatologist, following a detailed explanation to the patient. Treatment with Pregabalin started at a dose of 75 mg at bedtime while treatment with Duloxetine started at a dose of 30 mg a day (in the morning). After a period of 6 weeks patients were evaluated and dose was adjusted as necessary.

### Primary and secondary outcomes

The primary outcome of the study was subjective pain intensity evaluation using the visual analogue scale (VAS) [28]. Patients marked the extent of pain they had experienced during the previous week on a scale ranging between 0 and 100 (0 = no pain and 100 = worst pain).

The secondary outcomes include the following measures:

### Self-report questionnaires

Fibromyalgia—related symptoms questionnaires:

(1) Widespread pain index (WPI)—a count of the number of painful body regions. The WPI ranges from 0 to 19 [26]. (2) Fibromyalgia symptoms severity scale (SSS)–a measure of FMS major symptoms (fatigue, trouble thinking or remembering, waking up unrefreshed, pain or cramps in lower abdomen, depression, and headache). The SSS ranges from 0 to 12 [26]. Fibromyalgia diagnostic criteria are expressed as: WPI ≥ 7/19 and SSS ≥ 5/12, or WPI = 3-6/19, and SSS ≥ 9/12 (3). Fibromyalgia Impact Questionnaire (FIQ), designed to measure the components of health status most affected by FMS: physical functioning, ability to work, pain, fatigue, morning tiredness, stiffness, anxiety, and depression. FIQ scores range between 0 and 100 [29]. (4) Global pain scale (GPS) a screening tool for assessment of physical pain, affective effects of pain, specific clinical outcomes, and the degree to which the pain interferes with activities of daily living. GPS scores range between 0 and 100 [30].

Quality of life related questionnaire: (1) The RAND Health Status Survey, Short Form-36 (SF-36) was used to assess quality of life. The questionnaire evaluates physical functioning; bodily pain; role limitations due to physical health problems; role limitations due to personal or emotional health; general mental health; social functioning; energy/fatigue; and general health perception. Each index ranges between 0 and 100, with a high score indicating better health [31] (2). Medical Outcome Sleep Scale (MOS) provides assessment of several dimensions of sleep, including initiation, maintenance, respiratory problems, quantity, perceived adequacy, and somnolence. MOS scores range between 0 and 100 [32].

Psychological related questionnaires: (1) The Brief Symptom Inventory−18 (BSI-18) was used to evaluate psychological distress. The questionnaire generates a summary scale and three subscales: depression, anxiety, and somatization, and ranges between 0 and 24 [33]. (2) Beck Depression Inventory (BDI-II) consists of 21 items. Total scores range from 0 to 63, with higher scores indicating more severe depression [34].

Due to the subjective nature of pain, and the complexity and diversity of FMS symptoms, we defined the following indexes for quantifying this complex constellation of symptoms more precisely: Pain index = mean [VAS, GPS, WPI] and FMS index = mean [FIQ, BSI, reversed (Social function score)].

### Pain threshold and conditioned pain modulation

Pressure pain threshold (PPT) was assessed using a handheld computerized pressure algometer with a circular 1 cm^2^ probe (AlgoMed, Medoc LTD, Israel). PPTs were measured three times at the upper trapezius muscle. The baseline pressure applied was 0 kPa, with incremental increases of 30 kPa per second, up to a maximal pressure of 1000 kPa. The participant was instructed to report when the sensation changed from pressure to pain, at which point the probe was removed. The average of the second and third measurements was used in further analyses. For evaluating conditioned pain modulation (CPM), The PPT test was repeated during immersion of the non-dominant hand in 10°C cold water. The CPM is the difference in mean pain intensity between the two tests (2^nd^ and 3^rd^ PPT trials average subtracted from the 2^nd^ and 3^rd^ trials average while hand was immersed). Thus, effective pain inhibitory mechanisms are represented by higher (positive) values [35, 36].

### Brain activity imaging

Brain single photon emission computed tomography (SPECT) was conducted using 925–1,110 MBq (25–30 mCi) of technetium-99m-methyl-cysteinate-dimmer (Tc-99m-ECD) at 40–60min post-injection, using a dual detector gamma camera (Symbia T, Siemens Medical Systems), equipped with high resolution collimators. Data was acquired in 3-degree steps and reconstructed iteratively with Chang method (μ = 0.12/cm) attenuation correction. Both pretreatment and post-treatment SPECT images were normalized to the median maximal brain activity in the entire brain and were then reoriented into Talairach space using Oasis Neurology—NeuroGam application (Segami Corporation, Columbia, MD, USA) to identify Brodmann cortical areas and to compute the mean perfusion in each Brodmann area (BA) [37].

### Statistical analysis

Continuous data are expressed as means ± standard deviations (SD). Two-tailed independent t-tests were performed to compare variables between groups when a normality assumption held according to a Kolmogorov-Smirnov test. Net effect sizes were evaluated using Cohen’s d method, defined as the improvement from baseline after pharmaceutical intervention, subtracted from the improvement after HBOT, divided by the pooled standard deviation of the composite score. Categorical data were expressed in numbers and percentages, compared by chi-square/Fisher’s exact tests. To evaluate HBOT’s effect, a repeated-measure ANOVA mixed-model was used to compare post-treatment and pre-treatment data. The model included time, group and the group-by-time interaction. In questionnaire analysis, a Bonferroni correction was used for multiple comparisons. In SPECT analysis, FDR correction was used for multiple comparisons. A value of p<0.05 was considered significant. Pearson’s correlations were performed between Pain test scores or SPECT activation changes and the change in questionnaire scores before and after treatment. Data analysis was performed using MATLAB R2021b (MathWorks, Natick, MA) Statistics and Machine Learning Toolbox.

### Sample size estimation

According to Initiative on Methods, Measurement, and Pain Assessment in Clinical Trials (IMMPACT) recommendations, pain intensity reductions of 30% to 50% or more are considered useful. Therefore, the estimated sample size was calculated based on a reduction of 50% in the HBOT group and a reduction of 20% in the medication group, with a standard deviation of 40% [38, 39]. Assuming a power of 80%, and 5% two-sided level of significance, a total of 58 participants would be required, 29 participants in each arm. Considering a dropout rate of 15% the total sample size required is 70. The actual dropout rate was 25%, therefore the maximum enrolment was increased to 80.

## Results

### Patient characteristics and randomization

**Fig 1:**
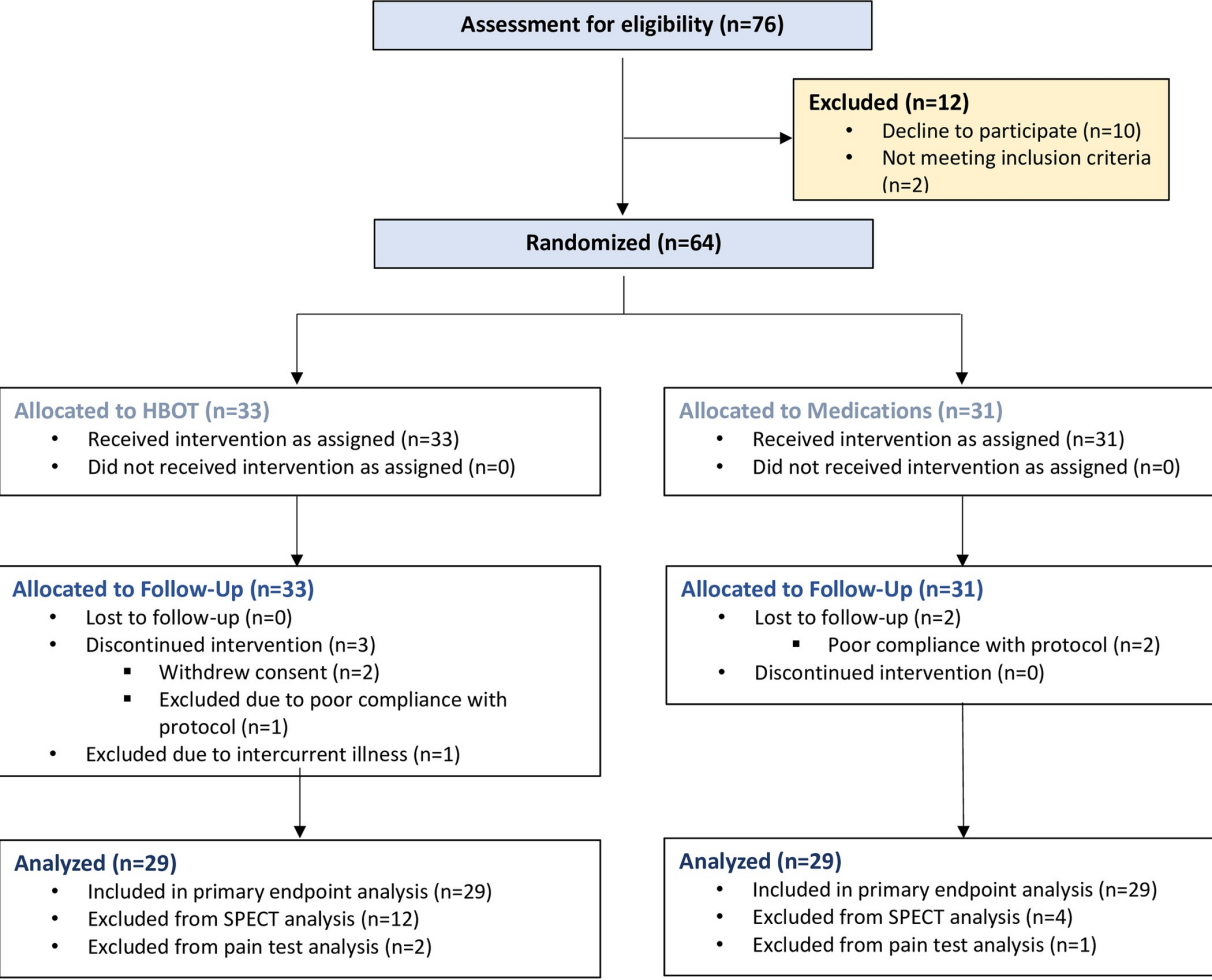
Study flowchart.

Seventy-six patients were eligible to participate in the study. Twelve patients were excluded before randomization. Thus, 64 were randomized to one of the two arms. Two patients from the HBOT group were excluded, one due to intercurrent illness, and one due to poor compliance and did not complete the post protocol assessments. Two additional patients withdrew their consent during treatment and did not complete evaluations. From the medication group, two patients were excluded due to poor compliance and did not complete the follow up assessments. Accordingly, 29 patients from the HBOT group and 29 patients from the medication group completed the protocol and were included in the analysis. The patient flowchart is presented in Fig 1. Patient baseline characteristics are detailed in Table 1. No statistically significant differences between the two groups were observed in baseline characteristics and symptoms.

**Table 1:** Baseline characteristics.

	HBOT group	Medication group
N	29	29
Age (years)	43.1±11.3	47.1±12.5
Male	8 (27.6)	4 (13.8)
Female	21 (72.4)	25 (86.2)
Years of education	14.1±2.6	14.4±2.4
Marital status		
Single	3 (10.3)	6 (20.7)
Married	16 (55.2)	15 (51.7)
Divorced	10 (34.5)	7 (24.1)
Widowed	0 (0.0)	1 (3.4)
Number of children	1.8±1.4	2.0±1.4
Employment status	19 (65.5)	16 (55.2)
Time from injury (years)	5.6±3.9	8.7±7.7
**Mechanism of injury**		
Motor vehicle accident—whiplash injury	21 (72.4)	21 (72.4)
Sever motor/ Motorcycle accident	3 (10.3)	3 (10.3)
Fall	3 (10.3)	3 (10.3)
Blow	1 (3.4)	1 (3.4)
Other	2 (6.9)	1 (3.4)
Diagnosis of FMS (years)	3.1±2.9	5.4±6.4
Disease related current chronic medications	10 (34.5)	9 (31.0)
Cannabis	6 (20.7)	5 (17.2)
Anti-depressant	5 (17.2)	5 (17.2)
Psychiatric treatment	10 (34.5)	10 (34.5)
Psychotherapy	15 (51.7)	14 (48.3)
Support group therapy	8 (27.6)	6 (20.7)
Previous suicide attempt	0 (0.0)	1 (3.4)

Data presented as n (%); continuous data, mean ± SD

### Primary outcome

**Fig 2:**
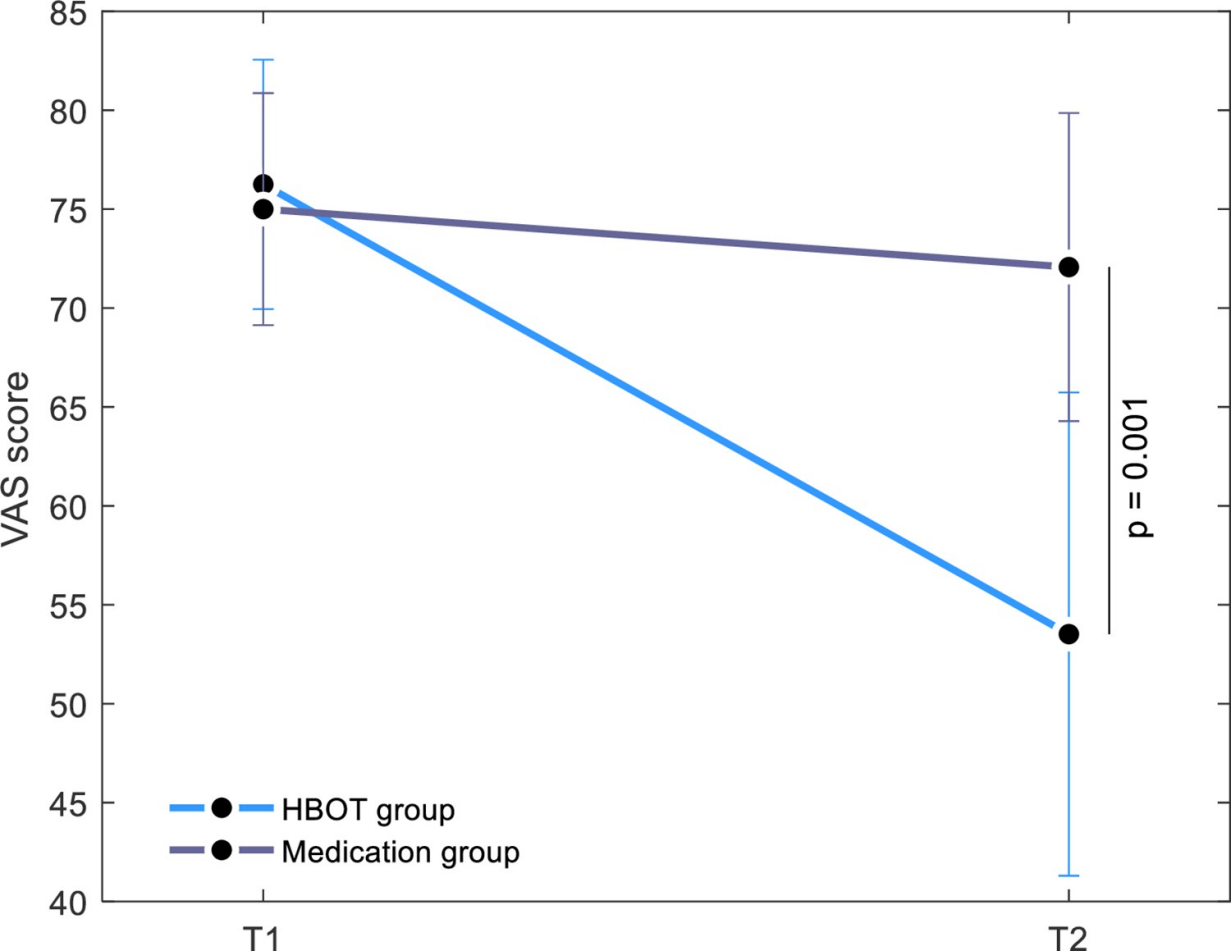
VAS score changes in the HBOT and medication groups. Model estimated marginal means and 95% confidence intervals. Significant three-months interaction effect, p = 0.001.

There was a significant group-by-time interaction in pain intensity evaluated using VAS score post-HBOT compared to the medication group, with a large net effect size (d = -0.95, p = 0.001) (Fig 2, Table 2).

**Table 2:** Questionnaire analysis.

	HBOT group				Medication group						ANOVA	
	PRE	POST	Change	Three months P-value	PRE	POST	Change	Three months P-value	P-value Baseline	Net effect size*	F	P-value
	29				29							
**Visual Analog Scale for Pain (VAS)**	76.3±16.0	53.5±30.3	-23.1±26.6	**0.0001**	75.0±15.1	72.1±19.8	-2.9±13.6	0.256	0.767	-0.96303	13.114	**0.001**
**WPI**	12.3±3.8	9.9±6.0	-2.4±5.9	0.0415	13.4±3.2	12.8±3.8	-0.6±2.9	0.258	0.235	-0.38268	2.086	0.154
**Symptom Severity Score (SSS)**	9.4±1.9	7.0±2.7	-2.4±3.0	**0.0003**	9.6±1.9	9.8±1.6	0.2±1.8	0.541	0.658	-1.04806	15.648	**0.000**
**Quality of Life SF-36**												
Physical functioning	35.5±19.1	52.0±27.6	16.4±19.1	**0.0001**	42.6±22.5	44.8±21.8	2.2±14.1	0.400	0.216	0.84724	10.226	**0.002**
Physical limitations	3.6±11.0	33.9±39.1	30.4±38.1	**0.0002**	2.6±10.1	10.3±21.3	7.8±23.2	0.083	0.730	0.71905	7.365	**0.009**
Emotional limitations	16.7±28.9	31.0±40.8	14.3±37.9	0.0561	19.5±32.2	24.1±40.0	4.6±31.8	0.442	0.729	0.27756	1.097	0.299
Energy	20.7±12.7	40.7±20.6	20.0±18.1	**0.0000**	17.8±14.5	20.9±14.0	3.1±12.8	0.204	0.425	1.08166	16.667	**0.000**
Emotional wellbeing	46.1±16.9	57.9±21.9	11.7±17.9	**0.0018**	42.1±18.5	41.0±19.4	-1.1±12.0	0.626	0.397	0.84356	10.137	**0.002**
Social function	27.7±20.7	51.3±26.6	23.7±23.9	**0.0000**	29.3±25.3	29.3±24.8	0.0±19.8	1.000	0.795	1.08086	16.643	**0.000**
Pain Domain	20.3±14.9	44.0±32.0	23.8±26.1	**0.0000**	18.3±15.6	21.6±17.7	3.3±12.8	0.178	0.630	1.0024	14.314	**0.000**
General Health Domain	37.5±17.5	45.0±23.3	7.5±16.8	0.0257	31.4±13.4	32.8±15.3	1.4±13.4	0.584	0.150	0.40327	2.317	0.134
**Physical Function Assessment (FIQ)**	66.8±13.4	47.2±24.7	-19.6±17.6	**0.0000**	70.1±13.9	66.6±16.0	-3.5±10.6	0.087	0.379	-1.10869	17.511	**0.000**
**Brief Symptoms Inventory BSI**												
Total	31.8±14.3	24.0±13.7	-7.8±10.7	**0.0006**	36.8±14.3	35.6±14.7	-1.3±8.2	0.409	0.197	-0.68817	6.746	**0.012**
Somatization	12.7±5.1	9.5±5.0	-3.2±3.3	**0.0000**	13.0±4.2	13.2±5.1	0.3±3.9	0.709	0.821	-0.95375	12.958	**0.001**
Depression	9.4±5.3	7.5±5.9	-1.9±4.9	0.0505	11.6±5.9	11.1±5.9	-0.5±3.6	0.442	0.161	-0.32198	1.477	0.229
Anxiety	9.7±5.8	7.0±4.8	-2.7±4.4	0.003	12.3±6.6	11.2±5.9	-1.0±3.1	0.080	0.128	-0.44473	2.818	0.099
**Beck Depression Inventory (BECK)**	29.6±11.8	22.9±11.3	-6.7±9.8	**0.0012**	24.9±11.7	23.8±13.5	-0.7±4.1	0.370	0.137	-0.80145	8.878	**0.004**
**Global pain scale (GPS)**	57.4±16.0	43.7±23.1	-13.8±15.8	**0.0001**	59.9±17.9	58.4±18.7	-1.5±10.7	0.453	0.588	-0.91024	11.803	**0.001**
**Medical Outcomes Study Sleep Scale MOS**												
Sleep Problems Index	53.6±9.5	58.0±10.1	4.4±9.5	0.0210	51.3±9.5	49.1±11.3	-2.2±10.8	0.281	0.384	0.64867	5.994	**0.018**
Quantity of sleep	5.7±2.0	5.9±1.7	0.3±1.7	0.4182	4.9±1.5	5.0±1.5	0.1±1.2	0.802	0.142	0.14471	0.298	0.587

Data are presented as mean ± SD; Bold, significant after Bonferroni correction

* Cohen’s d net effect size

** group-by-time interaction

### Secondary outcomes

Questionnaire analysis is summarized in Table 2 and S1 Table. At baseline, there were no significant differences in all domains between the groups. At baseline, all participants had positive fibromyalgia diagnosis criteria. After HBOT, 11 (37.9%) participants no longer met FMS diagnostic criteria, compared to none in the medication group.

Fibromyalgia related symptoms and pain questionnaires demonstrated significant improvements induced by HBOT. In both FIQ and GPS score, the HBOT group improved with a significant group-by-time interaction, and a large net effect size of (d = -1.11, p = 0.0001) and (d = -0.91, p = 0.001).

Regarding quality of life, the HBOT group improved in most health domains of the SF-36 questionnaire. Most significant domains were energy, social function, and pain with group-by-time significant interactions of (d = 1.08, d = 1.08, d = 0.11, p<0.0001). In the MOS, the HBOT group improved in sleep problems index, with a significant group-by-time interaction (d = -0.65, p = 0.018).

Improvements in psychological symptoms were also demonstrated after HBOT. In the BSI, the HBOT group improved in the total and the somatization scores, with a significant group-by-time interaction (d = -0.69, p = 0.012) and (d = -0.95, p = 0.001). Post-HBOT improvement was also found in the BDI score, with a significant group-by-time interaction and a large effect size (d = -0.80, p = 0.004). Correlations between all questionnaire domains is illustrated in Fig 5B.

**Fig 3:**
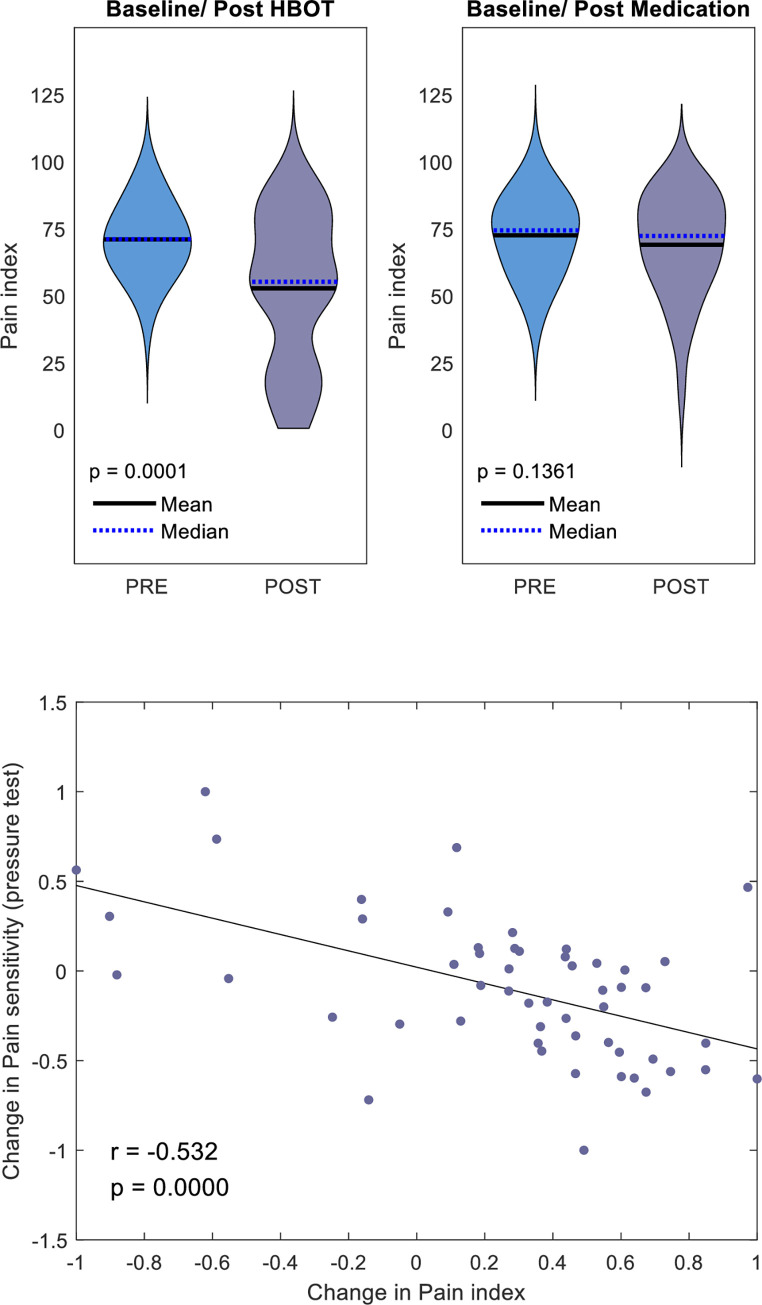
Change in questionnaire pain index. A. pain index changes shown in violin plots, B. Scatterplot of the change in pain sensitivity pressure test, and the change in pain index. r is Pearson’s correlation coefficient.

**Fig 4:**
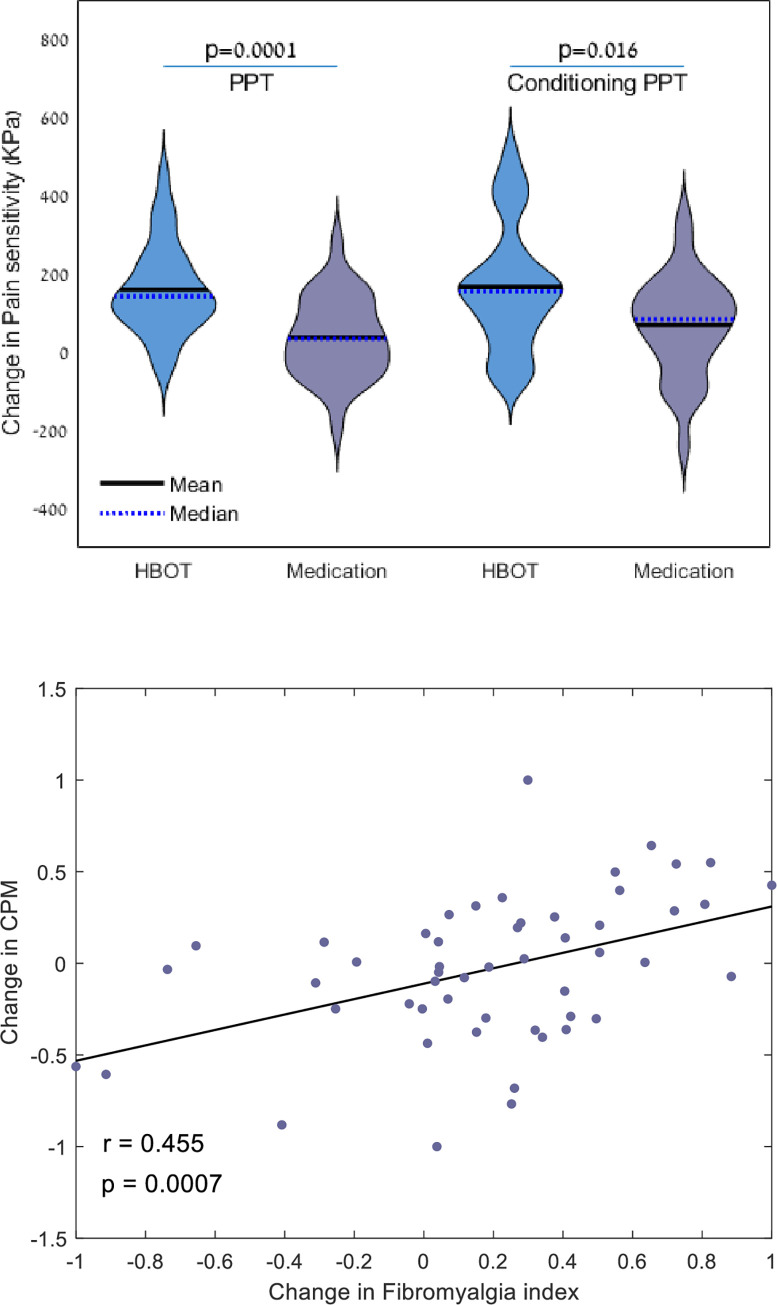
Change in pressure pain thresholds (PPT) at the upper trapezius muscle in the HBOT and medication groups. A. Left, pain sensitivity, Right, conditioned pain sensitivity (cold water). Data are presented in violin plot. B. Scatterplot of the change in conditioned pain modulation (CPM), and the change in FMS index. r is Pearson’s correlation coefficient.

Changes in PPT and CPM-PPT are illustrated in Fig 4A. In the HBOT group, PPT increased by 159.7±116.0 KPs with a significant group-by-time interaction, and a large net effect size of (d = 1.11, p = 0.0001). CPM-PPT increased by 175.5±161.8 with a significant group-by-time interaction, and a medium net effect size of (d = 0.72, p = 0. 016). In the medication group PPT increased by 37.3±104.0 KPs (p = 0.068), and the CPM-PPT increased by 69.7±129.6 KPs (p = 0.008). There was a significant negative correlation between pain index changes and changes in PPT analyzed in the entire cohort (r = -0.532, p<0.0001) as shown in Fig 3B. Pressure CPM showed no significant change between the groups, however CPM correlated with the change in FMS index (r = 0.455, p = 0.0007), as shown in Fig 4B.

**Fig 5:**
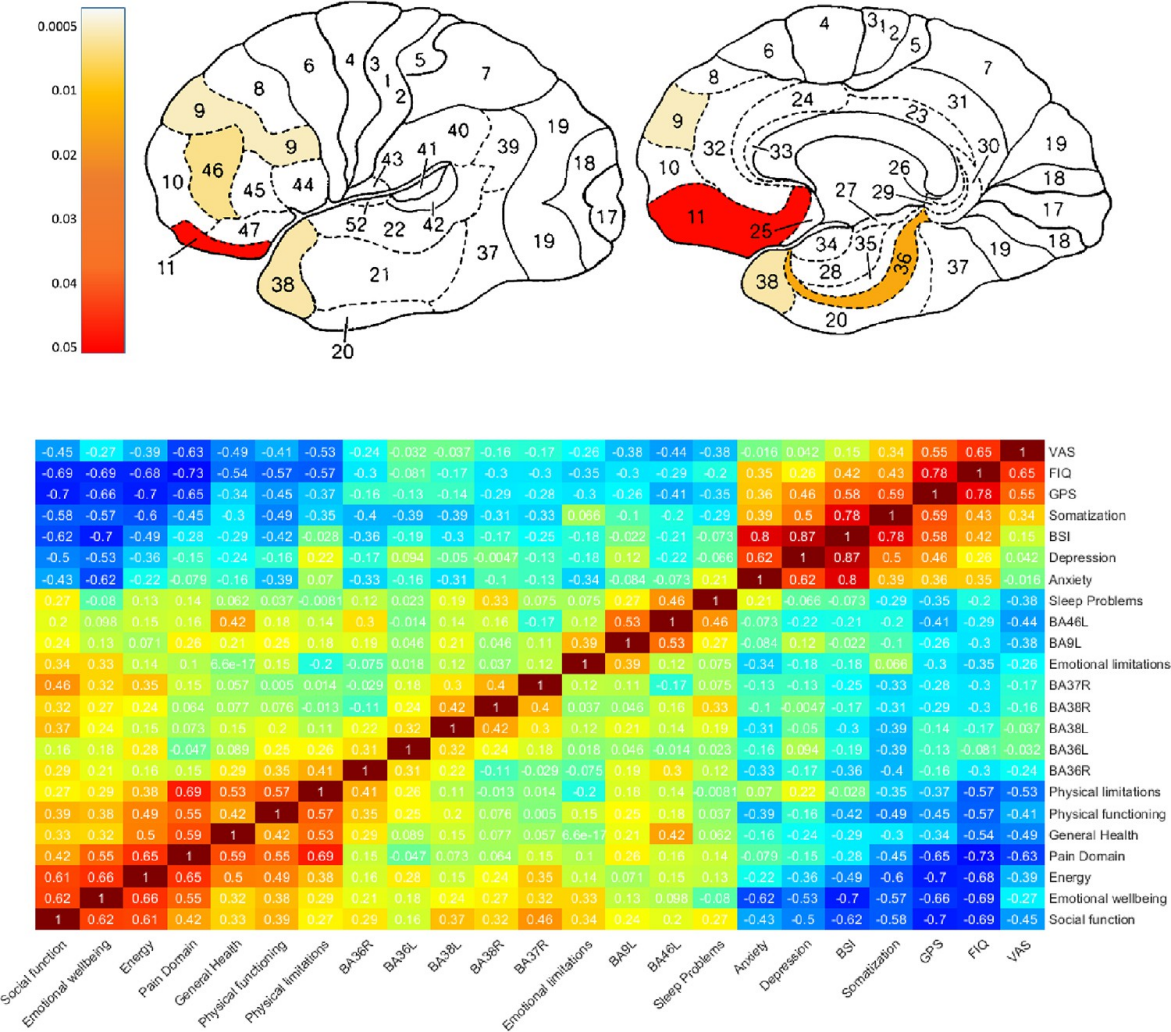
SPECT analysis. A. Increased post treatment activation in Brodmann areas (BAs) in HBOT group compared to Medication group. B. Pearson correlation coefficient heatmap representing changes in BAs activation and questionnaire scores. The parameters are ordered by hierarchical clustering using the Euclidian distance metric algorithm.

Seventeen participants in the treatment group and 25 participants in the medication group completed all brain SPECT evaluations. Sixteen participants underwent PET-CT imaging per the 1^st^ version of the protocol those were not included in the SPECT analysis. Increased post-treatment activation in Brodmann areas (BAs) is detailed in Table 3 and Fig 5A. Significant group-by-time interactions between the HBOT and the medication groups were demonstrated in the left frontal cortex—BA9, and BA46, and in the right temporal cortex—BA38 (FDR, p<0.05). Correlations between questionnaire domains and activation changes are illustrated in Fig 5B. Most significant correlations were found in the following areas: BA9L with VAS score, and SF-36/Emotional limitations (r = -0.38, 0.39, p = 0.02, 0.02); BA36R with SF-36/ Physical functioning, Physical limitations (r = 0.35, 0.41, p = 0.04, 0.01); BA36L, BA38L with BSI/Somatization score (r = -0.39, -0.40, p = 0.018, 0.021); BA38L with SF-36/Social function (r = 0.37, p = 0.03); BA46L with SF-36/General Health, GPS, MOS/Sleep Problems, and VAS (r = 0.42, -0.41, 0.46, -0.44, p = 0.011, 0.015, 0.005, 0.008).

**Table 3:** Increased post treatment activation in Brodmann areas (BAs)–SPECT analysis.

	HBOT group P-value	Medication group P-value	P-value Baseline	Net effect size*	F	P-value*
N	17	25				
BA 9 L	0.0002	0.222	0.18450	1.17	13.84	**0.0004**
BA 11 R	0.0471	0.988	0.82440	0.55	3.06	0.0471
BA 18 L	0.0262	0.522	0.43590	0.52	2.73	0.0975
BA 19 L	0.0028	0.194	0.42200	0.55	3.07	0.0743
BA 23 L	0.0247	0.885	0.62060	0.50	2.52	0.1100
BA 36 LEFT	0.0109	0.339	0.43560	0.58	3.38	0.0669
BA 36 RIGHT	0.0105	0.092	0.70540	0.77	6.01	0.0167
BA 37 RIGHT	0.0149	0.459	0.98510	0.28	0.76	0.3643
BA 38 LEFT	0.0007	0.486	0.23130	0.65	4.23	0.0415
BA 38 RIGHT	0.0003	0.137	0.27020	0.99	9.86	**0.0025**
BA 39 RIGHT	0.0088	0.595	0.89140	0.43	1.90	0.1608
BA 46 LEFT	0.0003	0.639	0.02630	0.97	9.46	**0.0033**

BA, Brodmann area; Bold

* ANOVA Group-by-Time Interaction FDR P<0.05

ROI activation is normalized to Brain Median; L, left, R, right

### Safety and side effects

Pharmacological treatment related side effects are provided in Table 4. The most common side effects were dizziness (58.6%), drowsiness/ weakness (20.7%), nausea (13.8%) and increased pain (10.3%). Other side effects include lack of improvement, decreased libido, dry mouth, palpitations, headache, and allergic reaction.

**Table 4:** Pharmacological treatment and side effects.

**Pharmacological treatment**	
Pregabalin (Lyrica)	22 (75.9)
Duloxetine (Cymbalta)	7 (24.1)
Medication adherence (weeks)	3.4±3.3
**Side effects**	
Dizziness	17 (58.6)
Drowsiness/ weakness	6 (20.7)
Nausea	4 (13.8)
Increased pain	3 (10.3)
Lack of improvement	3 (10.3)
Decreased libido	2 (6.9)
Dry mouth	2 (6.9)
Palpitations	1 (3.4)
Headache	1 (3.4)
Allergic reaction	1 (3.4)

Data presented as n (%); continuous data, mean ± SD

Eighteen (48.6%) participants in the HBOT group experienced side effects. Thirteen participants experienced mild middle ear barotrauma and one patient had sinus frontal pain. All resolved completely after taking a few days off treatment. Due to inability to equalized ear pressure, one patient underwent tympanic ventilation tubes insertion to continue protocol sessions, per his request to continue. One patient experienced temporary blurred vision during treatment session 25, which spontaneously resolved. Two patients reported tinnitus during HBOT, that spontaneously resolved within a few days. One patient reported an allergic reaction. All events were treated conservatively, and all participants completed their protocol.

## Discussion

The current study presents a prospective, active—control clinical trial, evaluating the effect of HBOT on FMS patients following Traumatic Brain Injury (TBI). We found that HBOT induced significant improvements in all FMS pain measures, quality of life, emotional and social function. The clinical changes were correlated with increased brain activity in the frontal and parietal regions, which extends previous findings and provides additional information regarding the etiology and treatment of FMS.

Chronic widespread pain is the central defining feature of FMS and thus pain intensity was chosen as the primary endpoint of the current study. The significant improvement in VAS—pain intensity documented in patients treated with HBOT compared to the standard of pharmacological care, implies a meaningful and significant effect on the core symptom of FMS. Although VAS is a subjective marker which has several limitations, it is considered the standard endpoint in pain—related disorders in general and in FMS studies in particular. While the effect on pain was evident in the primary endpoint, it was also evident in pain-related secondary outcomes, including the FMS diagnostic criteria, SF-36 pain domain and the GPS. Notably, the WPI significantly improved in the HBOT group compared to baseline, while no improvement was notice in the control group, however this improvement did not reach statistical significance on ANOVA analysis (Table 2). Since WPI measures pain spread rather than intensity, this may imply that the treatment is working at the CNS level to reduce pain threshold and not on specific on body location or may be the result of lack of statistical power for this secondary endpoint.

Highly significant improvement was observed following HBOT in outcome measures related to function, including the FIQ, which is the standard tool used for measuring the functional impact of FMS, as well as on the "Social function" component of the SF-36. The physical functioning and physical limitations components of the SF-36 were also significantly improved. Levels of energy and emotional wellbeing were found to be significantly increased as well. Moreover, a significant improvement was found in the Symptom severity scale (SSS), a tool which aggregates the non-pain related functional symptoms of FMS such as fatigue, dyscognition, abdominal pain and others. Taken together, these findings indicate that the beneficial effects of HBOT in the population of post–TBI FMS patients extend well beyond pain–reduction *per se* and have the ability to bring about a significant improvement in function in general. This finding is of great importance in the context of treating a highly prevalent syndrome such as FMS, which frequently affects young individuals at early stages of their carriers, and which may carry a devastating functional effect on this large population of patients.

In the current study, we chose both pressure pain threshold (PPT) as well as Conditioned pain modulation (CPM) in order to evaluate the psychophysical effects of HBOT. While PPT has frequently been used in order to diagnose FMS, as well as in order to measure response to treatment [40, 41], this tool has limitations related inter alia to the effects of anxiety on the reported threshold [42, 43]. CPM on the other hand is a well–established tool for assessing endogenous pain modulation [44] and is analogous to the phenomenon of "Diffuse Noxious Inhibitory Control" (DNIC) in animal models [45]. Central sensitization of pain in FMS, which is currently redefined as nociplastic pain, has been associated with a reduced ability to achieve CPM [46] and this failure of inhibitory pain control is considered an important aspect of the pathophysiology of FMS [46]. While conditioned PPT (reflecting CPM) significantly increased in the patients treated with HBOT, a significant increase was also observed in the medication group (Fig 4), thus making this result difficult to interpret. A significant correlation was observed however between CPM and the Fibromyalgia index (Fig 4B). Further research is warranted into the effects of HBOT on CPM in FMS patients.

In the current study, functional neuroimaging was performed using brain—SPECT testing with ECD as a marker for brain activity. This method has been used extensively in previous research for evaluating brain activity and perfusion patterns in FMS and changes in response to treatment [22, 47, 48]. As shown in the results (Fig 5), we were able to document significant changes in brain perfusion in FMS patients post HBOT, and to correlate these changes with specific symptom domains. The correlation between metabolic brain imaging and FMS symptoms further supports the discussed central nervous system pathology responsible for the development of FMS. Moreover, it should be noted that the post treatment effects were evaluated more than 1 week after the last HBOT session. Thus, the beneficial effect of HBOT may be related to central neuroplasticity effects of HBOT, as demonstrated in the brain SPECT analysis. these results emphasize the potential neuroplasticity effects of HBOT in this setup and the ability to correlate neuroplastic changes with clinical outcomes.

SPECT imaging has been used extensively in previous research for evaluating brain activity and perfusion patterns in FMS, and treatment response [23, 47–49,]. In the current study, the most pronounced changes were observed in the frontal and temporal brain areas, which is consistent with our previous findings in FMS patients. Specifically, this analysis provides further evidence for the differences between sub-groups of FMS patients. While in FMS triggered by childhood sexual abuse (CSA), post treatment brain SPECT imaging demonstrated significant increased brain activity in the prefrontal cortex, orbital frontal cortex, and subgenual area (BAs 8, 9, 44, 45, 25) [24], in TBI patients post treatment brain increased activity was found in the anterior temporal, prefrontal cortex, and perirhinal cortex (BA 38, 28, 46, 10, 11, and 36) [16]. Our results present characteristics of both diagnoses, which emphasize the important role of the central nerves system in the etiology of FMS.

Several limitations are worthy of mention regarding the current study. First, we do not have data regarding the long-term effects of HBOT treatment in our patient population. FMS is considered a chronic disorder, and while the effects of HBOT appear to be robust, further information will be necessary regarding the long-term effects of treatment. Second, the HBOT protocol used in this study included 60 daily sessions of HBOT. This protocol was found to have a significant neuroplasticity effect in previous studies; however, it might be possible that different protocols can be used for different patients in order to optimize the outcome per patient and further studies are needed to address this possibility. Third, the comparison group was treated with FMS medications, rather than a sham controlled intervention. Lastly, as the neuroscience of FMS continues to unravel, significant insight is being gained through implementation of additional functional neuroimaging, including fMRI [50, 51], Magnetic Electroencephalography (MEG) [52] and Magnetic Resonance Spectroscopy [53]. These techniques allow researchers to shed light on functional brain connectivity patterns in FMS and may eventually lead to the development of a FMS—specific pain signature [54]. Obviously, it will be necessary to utilize some of these novel instruments to broaden our understanding regarding the effects of HBOT on nociplastic pain.

### In summary

HBOT can improve pain, quality of life, and function in TBI triggered fibromyalgia, while increasing brain activity in frontal and parietal regions.

## Supporting information

S1 Checklist CONSORT 2010 checklist of information to include when reporting a randomised trial*. (DOCX)

S1 Table Questionnaire scores analysis–repeated measures ANOVA (group-by-time). (DOCX)

S1 Appendix Detailed evaluated results of each patient. Legends for S1: SN- serial number, VAS- Visual analog scale for pain, PPT- Pressure pain threshold, CPM- conditioned pain modulation, WPI—Widespread pain index, SSS—symptoms severity scale, FIQ—Fibromyalgia Impact Questionnaire, BSI—Brief Symptom Inventory, BECK- Beck Depression Inventory, MOS—Medical Outcome Sleep Scale, GPS—Global pain scale.
(XLSX)

S1 File (DOCX)

S2 File (PDF)
