# Supplementary material for: Hyperbaric oxygen therapy compared to pharmacological intervention in fibromyalgia patients following traumatic brain injury: A randomized, controlled trial
Source: PLoS One. 2023 Mar 10;18(3):e0282406. doi: 10.1371/journal.pone.0282406 (PMC10004612; doi:10.1371/journal.pone.0282406)
Supplement: S1 Table — (DOCX) [file pone.0282406.s002.docx]

Hyperbaric oxygen therapy compared to pharmacological intervention in fibromyalgia patients following traumatic brain injury: A, randomized, controlled trial

| **S1 Table: Questionnaire scores analysis – Repeated measures ANOVA (group-by-time)** | | | | | | |
| --- | --- | --- | --- | --- | --- | --- |
|  | **Main effect of group** | | **Main effect of time** | | **Interaction effect** | |
|  | **F** | **P-value** | **F** | **P-value** | **F** | **P-value** |
| **Visual Analog Scale for Pain (VAS)** | 2.26 | 0.139 | 23.17 | 0.000 | 11.25 | 0.001 |
| **Widespread pain index (WPI)** | 4.17 | 0.046 | 6.03 | 0.017 | 2.09 | 0.154 |
| **Symptom Severity Score (SSS)** | 11.09 | 0.002 | 11.00 | 0.002 | 15.65 | 0.000 |
| **Quality of Life SF-36** |  |  |  |  |  |  |
| Physical functioning | 0.00 | 0.994 | 17.71 | 0.000 | 10.23 | 0.002 |
| Physical limitations | 6.74 | 0.012 | 20.95 | 0.000 | 7.37 | 0.009 |
| Emotional limitations | 0.05 | 0.817 | 4.17 | 0.046 | 1.10 | 0.299 |
| Energy | 9.50 | 0.003 | 31.16 | 0.000 | 16.67 | 0.000 |
| Emotional wellbeing | 4.80 | 0.033 | 6.95 | 0.011 | 10.14 | 0.002 |
| Social function | 2.96 | 0.091 | 16.64 | 0.000 | 16.64 | 0.000 |
| Pain Domain | 5.92 | 0.018 | 24.94 | 0.000 | 14.31 | 0.000 |
| General Health Domain | 4.49 | 0.039 | 4.88 | 0.031 | 2.32 | 0.134 |
| **Fibromyalgia Impact Questionnaire (FIQ)** | 6.83 | 0.012 | 36.10 | 0.000 | 17.51 | 0.000 |
| **Brief Symptoms Inventory (BSI)** |  |  |  |  |  |  |
| Total | 5.23 | 0.026 | 13.03 | 0.001 | 6.75 | 0.012 |
| Somatization | 2.77 | 0.102 | 9.19 | 0.004 | 12.96 | 0.001 |
| Depression | 3.86 | 0.055 | 4.53 | 0.038 | 1.48 | 0.229 |
| Anxiety | 5.34 | 0.025 | 14.03 | 0.000 | 2.82 | 0.099 |
| **Beck Depression Inventory (BECK)** | 0.47 | 0.498 | 13.61 | 0.001 | 8.88 | 0.004 |
| **Global pain scale (GPS)** | 3.19 | 0.079 | 18.36 | 0.000 | 11.80 | 0.001 |
| **Medical Outcomes Study Sleep Scale (MOS)** |  |  |  |  |  |  |
| Sleep Problems Index | 5.41 | 0.024 | 0.66 | 0.419 | 5.99 | 0.018 |
| Quantity of sleep | 4.02 | 0.050 | 0.69 | 0.410 | 0.30 | 0.587 |
