# Supplementary material for: Hyperbaric oxygen therapy compared to pharmacological intervention in fibromyalgia patients following traumatic brain injury: A randomized, controlled trial
Source: PLoS One. 2023 Mar 10;18(3):e0282406. doi: 10.1371/journal.pone.0282406 (PMC10004612; doi:10.1371/journal.pone.0282406)
Supplement: S1 File — (DOCX) [file pone.0282406.s004.docx]

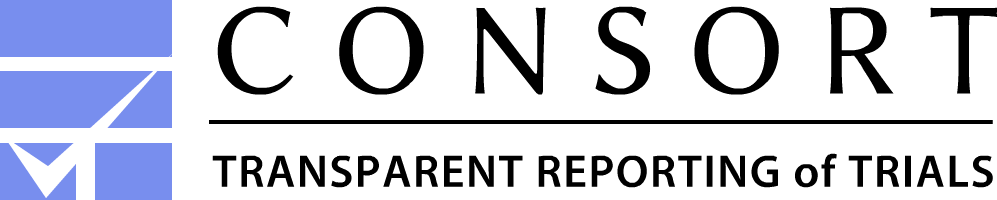


**CONSORT 2010 Flow Diagram 058-17-ASF**

Lost to follow-up (give reasons) (n=0)

Discontinued intervention (n=4)

- - Withdrew consent (n=2)
  - Poor compliance with protocol (n=1)
  - Excluded due to intercurrent illness (n=1)

Analysed (n=29)
♦ Excluded from analysis (give reasons) (n=0)

## Analysis

Analysed (n=29)
♦ Excluded from analysis (give reasons) (n=0)

Lost to follow-up (Poor compliance with protocol) (n=2)

Discontinued intervention (give reasons) (n=0)

## Follow-Up

## Enrollment

Allocated to intervention - HBOT (n=33)

♦ Received allocated intervention (n=33)

♦ Did not receive allocated intervention (give reasons) (n=0)

## Allocation

Allocated to intervention - medications (n=31)

♦ Received allocated intervention (n=31)

♦ Did not receive allocated intervention (give reasons) (n=0)

Randomized (n=64)

Excluded (n=12)

♦  Not meeting inclusion criteria (n=2)

♦  Declined to participate (n=10)

♦  Other reasons (n=0)

Assessed for eligibility (n=76)
