# Supplementary material for: Hyperbaric oxygen therapy compared to pharmacological intervention in fibromyalgia patients following traumatic brain injury: A randomized, controlled trial
Source: PLoS One. 2023 Mar 10;18(3):e0282406. doi: 10.1371/journal.pone.0282406 (PMC10004612; doi:10.1371/journal.pone.0282406)
Supplement: S2 File — (PDF) [file pone.0282406.s005.pdf]

<table>
<tr><td>Investigational Product
Hyperbaric chamber</td><td rowspan="2">Short Title:

Hyperbaric Oxygen Therapy for Fibromyalgia Syndrome</td><td>Protocol No.
058-17-ASF</td></tr>
<tr><td>Phase:
Investigator Initiated Phase III study</td><td>Version, Date
Ver.4.0, 10 OCT, 2017</td></tr>
</table>

CLINICAL STUDY PROTOCOL

Hyperbaric Oxygen vs. Standard Pharmaceutical Therapies for Fibromyalgia Syndrome - Prospective, Randomized Crossover Clinical Trial

Principal Investigators: Prof. Shai Efrati, MD, Head of the Sagol Center for Hyperbaric Medicine and Research, Shamir Medical Center

Date and Version Protocol 10 October 2017, Version 4.0

Statement of Compliance
This clinical trial will be conducted according to the current revision of the Declaration of Helsinki. It will be conducted in compliance with this protocol, with good clinical practice (CPMP/International Conference on Harmonization [ICH]/135/95), and with local laws and regulations relevant to the use of new therapeutic agents in the country of conduct.

Principal Investigator Signature Page

By signing below, I, the investigator approved the protocol and agree to conduct the clinical trial according to all stipulations of the protocol as specified in both the clinical and administrative sections.

I agree to comply with the ICH-GCP, European directive 2001/20/EC, applicable local law, and World Medical Association Declaration of Helsinki 2013.

Shai Efrati		*10.10.2017*
Name	Signature	Date

Shamir (Assaf Harofeh) Medical Center	Zerifin, Israel
Institution	City, Country

Log of Changes

Date	Description	By	Revision
07.03.2017	Original document	SE	1.0
07.03.2017	Appendix documents	SE	2.0
05.09.2017	Change of sub-investigators<br>Change 1.5ATA HBOT to 2ATA HBOT	SE	3.0
10.10.2017	Change PET-CT to SPECT	SE	4.0

Table of Contents

TABLE OF CONTENTS 4

LIST OF ABBREVIATIONS 6

1. INTRODUCTION 7

1.1 FMS definition and etiology 7
1.2 HBOT 8

2. STUDY OBJECTIVES 10

3. METHODS 11

3.1 Study population 11
3.2 Study protocol 12

3.2.1 HBOT protocol 12
3.2.2 Pharmaceutical protocol 12
3.2.3 Crossover 12

4. STUDY ENDPOINTS 13

4.1 Primary endpoint 13
4.2 Secondary endpoint 13

4.2.1 Fibromyalgia syndrome symptoms and quality of life questioners 13
4.2.2 Cognitive functions 13
4.2.2.1 Mindstreems cognitive battery test 13
4.2.2.2 CANTAB cognitive battery test 15

4.2.3 Brain imaging 16
4.2.3.1 Perfusion MRI+DTI 16
4.2.3.2 Brain SPECT 17

4.2.4 Brain Network Activation (BNA™) Analysis 17
4.2.5 Pain threshold and conditioned pain modulation (CPM) 19
4.2.5.1 Pain evaluations 20

4.2.6 Physical activity and Exercise capacity 21
4.2.6.1 Monitoring 24/7 physical activity 21
4.2.6.2 Exercise capacity 21

4.2.7 Mitochondria function 21
4.2.7.1 Mitochondrial oxygen consumption 22
4.2.7.2 Measurement of mitochondrial potential 22

4.2.8 Immune system 22
4.2.8.1 Inflammatory cytokines 22
4.2.8.2 Lymphocytes distribution 23
4.2.8.3 Lymphoproliferative response 23
4.2.8.4 NK cytotoxicity 23

4.2.9 Microbiome 24
4.2.9.1 Microbiome and human behavior: 24

4.2.9.2 Gut-brain signaling: 25
4.2.9.3 Microbiome evaluation method: 25

5. ADMINISTRATION AND REGULATION 27

5.1 Informed Consent 27
5.2 Confidentiality 27
5.3 Study Files 27
5.4 Randomization and handling of the control group 27
5.5 Adverse Events 28

6. STATISTICAL CONSIDERATIONS 29

6.1 Analysis Sets 29

6.1.1 Safety Analysis Set 29
6.1.2 Primary Efficacy Analysis Set 29
6.1.2.1 **Sample Size Considerations** 29

7. REFERENCES 30

List of Abbreviations

AE	Adverse events
ATA	Atmospheres
BNA™	The Brain Network Activation
CANTAB	Cambridge Neuropsychological Test Automated Battery
CBF	Cerebral blood flow
CHEPs	Contact Heat Evoked Potentials
CNS	Central Nervous System
CPET	Cardiopulmonary exercise test
CPM	Conditioned Pain Modulation
DMN	Default Mode Network
DTI	Diffusion tensor imaging
ERPs	Event Related Potentials
FDG	Fluorodeoxyglucose
fMRI	functional Magnetic Resonance Imaging
FMS	Fibromyalgia Syndrome
HBOT	Hyperbaric Oxygen Therapy
MoCA	Montreal Cognitive Assessment
mTBI	mild Traumatic Brain Injury
NRS	numeric rating scale
PET-CT	(combined) Positron Emission Tomography - Computed Tomography
PPCS	Persistent Post-Concussion Syndromes
PTSD	Post-Traumatic Stress Disorder
rsfMRI \ R-fMRI	resting state fMRI
SPECT	Single-Photon Emission Computerized Tomography
SSS	Symptom Severity Score
WPI	Widespread Pain Index

1. Introduction

1.1 FMS definition and etiology

FMS is a condition characterized by chronic widespread pain and diffuse tenderness, along with symptoms of fatigue, non-restorative sleep and cognitive difficulties [1, 2]. Over recent years, significant progress has been made regarding the pathogenesis of FMS. Currently, FMS is considered to represent a prototype of central sensitization, i.e. a condition characterized by an increase in the transmission and processing of pain within the central nervous system [3, 4]. The concept of central sensitization has subsequently been applied to a spectrum of overlapping "functional" conditions (e.g. Irritable Bowel Disorder -IBS, Temporomandibular Joint Disorder - TMJD etc), and has been supported and upheld by converging lines of evidence. On the one hand, psychophysical pain testing has clearly demonstrated phenomena such as a decrease in CPM (previously referred to as Diffuse Noxious Inhibitory Control, or DNIC) in many FMS patients [5], indicating a reduced capacity of the CNS to inhibit pain in these individuals. On the other hand, starting over a decade ago with the findings of Graceley et al [6], fMRI has been utilized in order to demonstrate the increased response of pain processing areas within the brain to experimental pain stimulation, among FMS patients. More recent functional imaging paradigms have uncovered additional layers of information regarding the nature of CNS central sensitization in FMS, particularly regarding the phenomenon of *increased connectivity* between particular brain areas in these individuals. Thus, Napadow et al have described an increase in connectivity between the DMN and the insula in FMS [7] as well as an association between decreased connectivity and reduced clinical pain [8]. Recent evidence has hinted at the possibility, that this increased connectivity may be modified by treatment modalities which bring about a clinical improvement in FMS, both pharmacological [9] and non – pharmacological [10]. Thus, neuro-plasticity appears to be a feasible concept in FMS, with direct relevance for the lynchpin of the FMS pathogenesis – the central sensitization paradigm. This understanding opens the window to a variety of possible interventions aimed at achieving the neuro- plasticity goal.

1.2 HBOT

HBOT is a well- known and reliable therapeutic modality, which has been implemented in a variety of clinical conditions. HBOT, the application of hyperbaric pressure in conjunction with increased oxygen content, has been shown in several clinical studies to have the capacity to induce neuroplasticity in injured brains even years after an acute insult [11-17]. HBOT induces neuroplasticity by stimulating cell proliferation [18], promotes neurogenesis of endogenous neural stem cells [19], regenerates axonal white matter [20], improves maturation and myelination of injured peripheral and cranial neural fibers [21, 22], induces brain angiogenesis[12], and stimulates axonal growth thus increasing the ability of neurons [23, 24]. To date, two prospective randomized controlled trials have demonstrated the efficacy of HBOT in fibromyalgia [25, 26]. The improvement was demonstrated in all aspects of FMS including pain threshold, fatigue, distress and quality of life [25, 26].

A growing amount of evidence has been accumulating over recent years regarding the efficacy of HBOT in the treatment of PPCS such as those caused by mTBI[27], which is defined as alteration of brain function caused by external forces, with one or more of the following: loss of consciousness for a duration of 0–30 minutes, posttraumatic amnesia for a duration of less than 24 hours, and Glasgow Coma Scale grade of 13–15 [28]. Specifically, HBOT appears to be capable of inducing cerebral angiogenesis, thus improving perfusion to chronically damaged brain tissue even months to years after the injury [29]. Thus, as recently reviewed by Figueroa et al, 5 out of 5 peer-reviewed clinical trials have demonstrated therapeutic effects of HBOT on symptoms of mTBI/PPCS as well as PTSD [30].

One possible mechanism explaining the neuro-plasticity potential of HBOT relates to the effect on the mitochondrial function of Glia cells [31], a cell population which is postulated to play an integral role in the pathogenesis of central sensitization and chronic pain [32, 33]. Breathing oxygen under hyperbaric conditions has the capacity to significantly increase brain oxygen tension, increasing tissue oxygenation and oxygen delivery into Glial mitochondria [34, 35].

The etiology of FMS is considered to be multifactorial. A genetic underpinning is widely assumed [36] but is most probably polygenic, similar to other complex human disorders. In addition, various triggers have been associated with the fibromyalgia syndrome, including (but not limited to) physical trauma [37], such as whiplash injury [38] and head injury, infection (e.g. EBV) [39] as well as stress, both acute and chronic. Notably, in some cases a specific trigger such as a motor vehicle accident can easily be elicited in the history as occurring in close proximity to the development of symptoms, while in other cases no such specific event can be identified.

We have previously studied the efficacy of HBOT as a treatment for FMS in a prospective, active control, crossover clinical trial [25]. In our previous study, 60 female patients underwent 40 sessions of HBOT (90 minutes per session). The results demonstrated significant amelioration of all FMS symptoms, with significant improvement in life quality; furthermore, we were able to demonstrate significant neuroplasticity on SPECT imaging, with a decrease of the hyperactivity in posterior regions and elevation of the reduced activity in frontal areas.

2. Study Objectives

In the proposed study, we intend to both repeat and expand our previous findings, treating FMS patients with HBOT while performing an extensive batter of evaluation both before and after treatment. Our goal is to both establish the role of HBOT in the treatment of FMS as well as to study in depth the mechanisms of neuroplasticity involved and the effect of HBOT. Through extensive advanced imaging and laboratory investigation we also hope to shed significant additional light on the underlying pathogenesis of the fibromyalgia syndrome.

In the current study, we plan to compare HBOT to current standard of care of FMS (pharmacological and non – pharmacological). Notably, since HBOT is associated with a significant investment of time and resources (on behalf of both the patient as well as the health systems), one would need to demonstrate a decisive advantage of this method over the currently recommended standard of care in order to justify the associated investment.

In view of the above mentioned data regarding the efficacy of HBOT in the treatment of mTBI, we would like to focus our study on cases of FMS in which a clear physically traumatic trigger is evident.

3. Methods

3.1 Study population

The study will include 70 fibromyalgia patients in whom physical trauma, such mTBI, could be considered as the trigger for FMS. Each participant will be examined at the time of recruitment and a diagnosis of FMS will be verified, based on the updated 2016 diagnostic criteria [40]. These criteria are based on the use of the widespread pain index (WPI) and the Symptom severity Score (SSS).

Exclusion criteria will include the presence of systemic inflammatory disorders including inflammatory rheumatological and autoimmune disorders. Patients suffering from active malignancy, chronic ongoing infection and major psychiatric disorders (excluding anxiety) will be excluded. Patients currently or previously treated with Duloxetine (Cymbalta) or Pregabalin (Lyrica) will also be excluded.

In the current study we will recruit patients not currently being treated with medications specific for FMS, including SSRI, SNRI, gabapentanoids and tricyclics, opiods and medical cannabis. Patients who are on such treatment will be required to discontinue treatment 2 weeks before recruitment.

In addition, patients who have one of the following criteria will also be excluded: Patients will be excluded if they will have one of the following criteria: had been treated with HBOT for any other reason prior to their inclusion; Chest pathology incompatible with pressure changes (including active asthma); Inner ear disease; Claustrophobia; Inability to perform awake brain MRI test; Previous neurologic conditions (eg. Epilepsy, neuromuscular diseases, metabolic diseases, etc.); Brain tumors; Skull base fractures; Active malignancy; s/p neurosurgery that included: ventricular drainage, subdural hematomas drainage, epidural hematomas drainage, intracerebral hemorrhage evacuation. Depressed fracture surgery, (Patients suffering from Encephalomalacia per MRI imaging will not be excluded). Inability to provide informed consent.

3.2 Study protocol

Patients will undergo randomization upon recruitment to one of the two study groups. One group will proceed to a course of HBOT treatment while the second group will commence with standard treatment for FMS, as outlined in the Israeli guidelines for the diagnosis and treatment of FMS [41]. These patients will be given detailed education regarding the nature of FMS as well as recommendations regarding non – pharmacological interventions recommended for FMS, including graded physical exercise, hydrotherapy, movement-meditative treatments (e.g. Tai Chi) and cognitive behavioral treatment (CBT).

3.2.1 HBOT protocol

A total of 60 daily hyperbaric oxygen treatment sessions will be administrated 5 days per week. Each session will include exposure of 90 minutes to 100% at 2 ATA, with 5 minutes air breaks every 20 minutes.

3.2.2 Pharmaceutical protocol

Patients will be offered pharmacological treatment with one of the two medications currently licensed for the treatment of FMS in Israel, i.e. Cymbalta and Lyrica. Treatment with Lyrica will start at a dose of 75 mg at bedtime while treatment with Cymbalta will start at a dose of 30 mg a day (in the morning). After a period of 6 weeks patients will be evaluated and dose will be adjusted as necessary. Patients may also be switched from one medication to the other based according to clinical judgment.

3.2.3 Crossover

After 3 months of either pharmaceutical or HBOT, once the 2nd evaluation is completed, all patients in both groups will be offered to switch to the alternative treatment group.

4. Study endpoints

4.1 Primary endpoint

The primary end point of the study will be the measurement of daily pain on a (0-10) Visual analogue Scale (VAS) [42]

4.2 Secondary endpoint

The secondary end-points of the study will include the following:

4.2.1 Fibromyalgia syndrome symptoms and quality of life questioners

- Global Pain Scale (GPS) [43].
- Patient global impression of change (PGIC) [44]
- Fibromyalgia Impact Questionnaire (FIQ, Hebrew version) [45]
- Wide Spread Pain Index (WPI) and Symptom Severity Scale (SSS) [46]
- Quality of Life (SF-36) [47]
- Medical Outcome Sleep Scale (MOS) [48]
- Beck Depression Inventory (BDI-II) [49]
- Quality of life will be evaluated by the EQ-5D
- The Brief Symptom Inventory-18 (BSI-18) [50]

4.2.2 Cognitive functions

Cognitive functions will be evaluated by 2 test batteries: Mindstreems and CATAB.

4.2.2.1 Mindstreems cognitive battery test

The Mindstreams battery includes several cognitive tests devised to check various aspects of brain capabilities. In the current study we will evaluate the cognitive indices based on the scores of the 6 cognitive tests listed below, which are expected to be relevant for mTBI. For detailed description of all cognitive tests in Mindstreams battery see [50]. The tests are:

- ***Verbal memory:*** Ten pairs of words are presented, followed by a recognition test in which the first word of a previously presented pair appears together with a list of four words from which the patients choose the other member of the pair. There are four immediate repetitions and one delayed repetition after 10 min.
- ***Non-verbal memory.*** Eight pictures of simple geometric objects are presented, followed by a recognition test in which four versions of each object are presented, each oriented in a different direction. There are four immediate repetitions and one delayed repetition after 10 min.
- ***Go–No-Go test.*** In this continuous performance test, a colored square (red, green, white or blue) appears randomly on the center of the screen. The patient in then asked to respond quickly only for red squares by pressing the mouse button, and inhibit his reaction to any other colored square.
- ***Stroop test.*** Timed test of response inhibition modified from the Stroop paper-based test. In the first phase, patients choose a colored square matching the color of a general word (for example, the word "Cat" appears in red letters, the patient must choose the red square out of two colored squares in the following screen). In the next phase (termed the Choice Reaction Time test), the task is to choose the colored square matching the name of the color presented in white letter–color. In the final (Stroop interference) phase, patients are asked to choose the colored square matching the color and not the meaning of a former color-naming word, presented in an incongruent color (for example, the word "RED" appears in green letters, the patient is asked to choose the color green and not red, a task requiring the ability to inhibit an automatic response to the meaning of the word).
- ***Staged information processing test.*** Timed test requiring a reaction based on solving simple arithmetic problems (pressing right/left mouse button if the answer higher/lower than 4, respectively) with three levels of information processing load (single digit, two digits addition/subtraction and three digits addition/ subtraction problems), each containing three speed levels (3, 2, and 1 second for the presentation of the stimuli).

- ***Catch game***. A test of motor planning that requires participants to catch a falling object on a computer screen by moving a paddle horizontally so that it can "catch" the falling object.

To assign scores, Mindstreams data will be uploaded to the NeuroTrax central server. Outcome parameters will be calculated using custom software blind to diagnosis or testing site. To minimize differences related to age and education, each outcome parameter will be normalized and fit to an IQ-like scale (mean=100, STD=15) according to patient's age and education. We note that the score evaluation will be based on normative data from cognitively healthy individuals collected in controlled research studies that were part of more than 10 clinical sites [51].

4.2.2.2 CANTAB cognitive battery test

Patients' cognitive functions will be assessed by CANTAB computerized cognitive tests (Cambrdige cognition , England) [52]. The CANTAB is a semiautomated test battery which can be administered on a laptop PC and more recently has been modified for administration on a handheld tablet. The current release of CANTAB Eclipse comprises 25 tests designed to assess components of cognitive function which fall into 7 broad groups of tests: visual memory, executive function, working memory and planning, attention, semantic/verbal memory, decision making and response control, social cognition, and screening/familiarization.

CANTAB tests evaluate various aspects of brain functions and include: Memory tests (paired associated learning, delayed matching to sample, graded naming test, pattern recognition memory, spatial recognition memory, verbal recognition memory, spatial span, spatial working memory), Attention tests (reaction time, rapid visual information processing, choice reaction time, simple reaction time, match to sample visual search, attention switching task), Executive Function tests (one touch stockings of Cambridge, attention switching test, intra-extra dimensional set shift, spatial span, spatial working memory, stop signal task), Decision making tests (Cambridge gambling task, affective Go-No-Go, information sampling task). Cognitive index scores will be computed from normalized outcome parameters for these tests for memory, executive function,

attention, decision making, social cognition [53, 54]. A global cognitive score will be computed as the average of all index scores for each individual. Notably, the patients will be given three different test versions of the CANTAB test battery at baseline, 3 months control period and after HBOT, to allow repeated administrations with minimal learning effect. Test-retest reliability for those versions will be evaluated and found high, with no significant learning effect [55].

4.2.3 Brain imaging

Brain imaging will include 2 type of imaging: perfusion MRI+DTI including resting state functional MRI and Brain SPECT.

4.2.3.1 Perfusion MRI+DTI

The MRI protocol includes the following sequences: Axial T1, T2, FLAIR, Axial Diffusion weighted, Axial T1-PostGad, DSC, DCE, DTI, FLAIR,T1, T2 and SWI.
MRI scans sequences parameters:

- ***DSC:*** 50 T2*-weighted gradient-echo echo planar imaging (EPI) volumes will be acquired, 2 repetitions before a bolus injection of Gadolinium-DTPA (Gd-DTPA), 48 repetitions after injection of Gd-DTPA. Sequence parameters: TR=2,300 ms, TE= 40ms, flip angle = 30°, voxel size = 1.8 x1.8, Matrix = 128x128, No. of slices = 25, Slice thickness = 3.9 mm.
- ***DCE***: three T1 weighted Fast Low Angle SHot (FLASH) volumes will be acquired with different flip angles (2, 7 and 15 degrees) followed by 70 dynamic T1 weighted FLASH volumes with flip angle of 15 degrees after bolus injection of Gd-DTPA. Sequence parameters: before injection: TR=4.09, after injection - TR=4.86 ms, TE= 1.76 ms, Voxel size = 1.5x1.5, Matrix = 192X192, No. of slices = 26, Slice thickness = 3.5mm.
- ***DTI***: 30 diffusion weighted images will be scanned with different gradient directions (b=1000) and one volume without diffusion weighting, with the following parameters: TR=10,300 ms, TE=89 ms, Voxel size = 1.8X1.8, Matrix = 128 X 128, No. of slices = 63, Slice thickness = 2.2mm

- ***Resting state fMRI (rsfMRI\ R-fMRI)***: a method of functional brain imaging that can be used to evaluate regional interactions that occur when a subject is not performing an explicit task. This resting brain activity is observed through changes in blood flow in the brain which creates what is referred to as a blood-oxygen-level dependent (BOLD) signal that can be measured using fMRI.

4.2.3.2 Brain SPECT

Brain single photon emission computed tomography (SPECT) will be conducted using 925–1,110 MBq (25–30 mCi) of technetium-99m-methyl-cysteinate-dimmer (Tc-99m-ECD) at 40–60min post-injection using a dual detector gamma camera (Symbia T, Siemens Medical Systems) equipped with high resolution collimators. Data will be acquired in 3-degree steps and reconstructed iteratively with Chang method (μ=0.12/cm) attenuation correction.
Both pretreatment and post-treatment SPECT images will be normalized to the median maximal brain activity in the entire brain and will be then reoriented into Talairach space using Oasis Neurology - NeuroGam application (Segami Corporation, Columbia, MD, USA) to identify Brodmann cortical areas and to compute the mean perfusion in each Brodmann area (BA).

4.2.4 Brain Network Activation (BNA™) Analysis

BNA™ Analysis System is a software only device that utilizes advanced algorithms to analyze the brain network activity of the brain from the recorded EEG data (AMAR: 27860001). The BNA™ Analysis system provides both qualitative network maps of the activity as well as quantitative scores that can be used as a neuromarker for disease. Event Related Potentials (ERPs), which are temporal reflections of the neural mass electrical activity of cells in specific regions of the brain that occur in response to stimuli, may offer such a method, as they provide both a noninvasive and portable measure of brain function. The ERPs provide excellent temporal information, but spatial resolution for ERPs has traditionally been limited. However, by using high-density electroencephalograph (EEG) recording spatial resolution for ERPs is improved

significantly [56-59]. The paradigm for the current study will combine neurophysiological knowledge with mathematical signal processing and pattern recognition methods (BNA™) to temporally and spatially map brain function, connectivity and synchronization.

The following parameters will be evaluate by BNA™ technology:

- ***Visual GO/NO-GO*** - The Go/No-Go paradigm is among the most well-established tests of response inhibition to perceptual stimuli. The task involves sensory processing, motor activity (a response) and response inhibition. In this task, subjects see a series of alphabetical letters and are required to react according to the letters. Letters are presented every 2 seconds on average. 80% of the stimuli are the letters B, C, D, E, F or G providing the Go cues ("Sensory”), to which subjects are asked to respond as quickly as possible (“Motor”), and 20% are an “X” providing the No-Go cue ("Response inhibition”), that require subjects to inhibit their response. The stimuli are presented on the center of a black background monitor for 150 ms and are located between two vertical white lines which remains constant throughout the task. The inter stimulus interval varies randomly between 1000 ms and 2600 ms with steps of 250 ms. The total number of trials is 400. Task duration is ~14 minutes.
- ***Auditory Oddball*** - The auditory oddball task is a classic EEG paradigm that has been studied extensively and used in a variety of neurological patient populations. The task involves executive functions, attention and memory processes. In this task, subjects hear a series of auditory tones and are required to react according to the tones. Tones are presented once every 1.5 seconds on average. A total of 80% of the sounds ("Sensory”) are 2000 Hz tones. A total of 10% of the sounds ("Memory") are 1000 Hz tones to which participants respond by pressing a button. The remaining 10% of sounds ("Attention") are complex sounds.
- ***Sensory Evoked Potential*** - Contact heat stimuli were delivered to the right, dominant proximal volar forearm using a round thermode of 572.5 mm2 (PATHWAY, Medoc Ltd., Ramat-Yishai, Israel) in four-block heat stimulation sessions to produce Contact heat evoked potential (CHEPs). Each CHEPs stimulus

trial began with the thermode applied to the skin, followed by the triggering of the heat stimulus, and ended with a beep, which prompted subjects' to rate the pain produced by the heat stimulus. This thermode application was repeated in a clockwise manner on the designated area of the forearm. On Visit 1, subjects underwent a temperature-determination protocol, in which they received three different heat stimuli of the same temperature, ranging from 38°C to 52°C. Subjects were instructed to rate each stimulus on a numeric rating scale (NRS), where 0 indicated the absence of pain and 10 indicated the worst imaginable pain. The two temperatures which subjects rated as 3 and 6 on the NRS were chosen as the low and high-temperature, respectively, used during proceeding Pre and Post HD-tDCS EEG recordings. The first two blocks of heat stimuli used the low-temperature determined (NRS rating of 3) for each individual subject and were separated by a 5 minute interval. The following two blocks of heat stimuli used the high-temperature determined (NRS rating of 6) for each individual subject and were also separated by a 5-minute break. The low and high-temperature blocks were further separated by a 15-minute interval between them. There were 20 stimuli (trials) per block and the inter-stimulus interval ranged from 8 to 13 sec.

- ***Resting States*** - EEG will be recorded at rest (no cognitive task), several minutes with eyes open and several minutes with eyes closed (between 2 and 5 min). The aim of the resting EEG recording is to extract patterns of the DMN. The DMN is a network comprising many brain regions which show in fMRI studies reduced activation during task execution. Recently, the DMN has been studied also in EEG signals (Laufs et al. 2003).

4.2.5 Pain threshold and conditioned pain modulation (CPM)

Pressure pain threshold (PPT) will be assessed using a handheld computerized pressure algometer with a circular 1 cm2 probe (AlgoMed, Medoc LTD, Israel). PPTs will be measured three times at the upper trapezius muscle. The baseline pressure applied will be 0 kPa, with incremental increases of 30 kPa per second, up to a maximal pressure of 1000 kPa. The participant will be instructed to report when the sensation changed from

pressure to pain, at which point the probe will be removed. The average of the second and third measurements will be used in further analyses. Decreased inhibitory conditioned pain modulation (CPM) effectiveness has been observed in FMS patients (*de Souza JB et al. The Clinical journal of pain 2009; Julien N et al. 2005; Kosek E et al. 1997; Lautenbacher S, et al. 1997; Paul-Savoie E et al. 2012*). For evaluating CPM, The PPT test will be repeated during immersion of non-dominant hand to 10 degrees cold water. The CPM is the difference in mean pain intensity between the tests. Thus, effective pain inhibitory mechanisms are represented by higher (positive) values *(Philippe Chalaye et al. 2013).*

During the pain intensity evaluation (CPM efficacy) brain cortical activity will be continuously monitored using the EEG -DELPhI system (Amar: 570406) evaluating the cortical manifestation of the chronic pain and its correlation with the evaluated CPM efficiency.

4.2.5.1 Pain evaluations

Participants evaluated pain intensity with a computerized numeric pain rating scale (CNPRS) ranging from 0 (no pain) to 10 (most intense pain tolerable).

- **Heat pain threshold and heat pain tolerance -** Heat pain threshold is determined as the minimum temperature causing pain and heat pain tolerance is established as the highest tolerable temperature.

  Thermal pain is induced with thermal electrode (thermode). Thermode temperature will initially be set at 32.0°C and gradually increase at a rate of 0.3°C/sec. Participants will be instructed to report when the sensation produced by the thermode changed from heat sensation to pain (heat pain threshold) and when the pain became unbearable (heat pain tolerance). This procedure will be conducted twice for every subject and the mean of the two trials will be calculated. The thermode will be placed on adjacent areas of the forearm for every trial to avoid primary skin hyperalgesia.
- **Heat test-stimulus (HTS) -** Following heat pain thresholds assessments, the thermode will be applied on the left forearm for 120 seconds at constant temperature. The temperature will be individually adapted to induce a mean pain intensity of 60/100 based on heat pain threshold and tolerance values using the following

formula: Heat pain threshold + ((heat pain tolerance - heat pain threshold)/2) (*Chalaye P et al. 2013*). Participant's pain intensity will be evaluated by CNPRS. If the pain intensity produced by this temperature will not induce an average pain of 60/100, the temperature will be adjusted accordingly. The 120-second HTS will be done before and after the CPT using the same thermode temperature. The thermode will be placed on adjacent areas of the forearm for every HTS trial to avoid primary skin hyperalgesia.

- **Cold Pressor Test (CPT)** - Participants immerse their dominant arm (up to the elbow) in circulating cold (12°C) water for 2 minutes. Participants will be instructed not to move or contract their arm during the immersion. Pain intensity ratings will be provided with the CNPRS automatically every 20 seconds during the entire 2-minute immersion period.

4.2.6 Physical activity and Exercise capacity

4.2.6.1 Monitoring 24/7 physical activity

The daily physical activity will be objectively tracked by FitBit watch technology. The FitBit watch will be also wired during night for measurements of the time asleep, restless and awake, Fitbit trackers help you understand each night to make the most of each day

4.2.6.2 Exercise capacity

Participants will undergo exercise testing using a modified Balke treadmill protocol and continuous expired gas analysis. Resting and exercise vital signs will monitored continuously. The exercise duration and exercise-limiting symptoms will be recorded. The peak VO2, VCO2 and respiratory exchange ratio (RER) will be averaged over the last 15 seconds of the exercise test. The ventilatory equivalent (VE/VCO2 slope) will be calculated from start of exercise to the end of exercise.

4.2.7 Mitochondria function

4.2.7.1 Mitochondrial oxygen consumption

To monitor the consumption of oxygen and extracellular acidification in intact adherent lymphocytes, Seahorse Bioscience XFe96 Extracellular Flux Analyzer will be used (Seahorse Bioscience, North Billerica, MA, USA). For these experiments, lymphocytes will be seeded to 3 × 104 cells/well in 12 wells (for each cell line) of XFe 96-well cell culture microplate (Seahorse Bioscience) in 200 µL of DMEM and incubated overnight (14–16 h) at 37 °C in 5% CO2 atmosphere. After replacing the growth medium with 175 µL of bicarbonate-free DMEM pre-warmed at 37 °C cells will be preincubated at 37 °C for 1 h for degassing before starting the assay procedure. Oxygen consumption rate (OCR) and extracellular acidification rate (ECAR) will be measured using mix/wait/measure times of 3/3/3 min. After baseline measurements of OCR and ECAR, OCR will be measured after sequentially adding to each well 25 µL of oligomycin (1 µM), carbonyl cyanide-ptrifluoromethoxyphenylhydrazone (FCCP) (0.25 µM), and Rotenone/antimycin A (1 µM) and ECAR by glucose (10 µM), oligomycin (2 µM) and 2-deoxyglucose (100 µM) to the indicated final concentrations using the included ports on the XFe96 cartridges. Further analysis of these experiments will be performed as described [12]. Metabolic profile of the individual cell lines will be generated by plotting OCR against ECAR.

4.2.7.2 Measurement of mitochondrial potential

Cells will be incubated with 3–3′ dihexyloxacarbocyanine iodide [DiDOC6 (3)] (40 nm in Phosphate buffer saline +30 µg mL–1 Propidium iodide) (Molecular Probes, Eugene, OR, USA) for 15 min at 37 °C in the dark. Propidium iodide will be added to gate out dead cells. Cells will be transferred immediately to a tube on ice for analysis of the fluorescence intensity by flow cytometry (excitation 488, emission 525 nm)

4.2.8 Immune system

4.2.8.1 Inflammatory cytokines

Blood Tests will include: IL-1, IL-6, Tumor necrosis factor-alpha, CRP.

4.2.8.2 Lymphocytes distribution

Using a 4-color FACS the following tests will be performed: CD4 number, CD8 number, CD4:CD8 ratio, CD8+CD28null, Naïve B-Cells number, CD4CD25 positive number, NK numbers.

Isolation of peripheral blood mononuclear cells (PBMC) will be isolated using density gradient centrifugation and will be held 3 times in 1640 RPMI medium. Cells will be frozen in freezing medium and kept in liquid nitrogen until thawed for analysis.

PBMCs will be held twice in PBS with 3% FCS. 1X10^5 cells will be re-suspended in 100 microliter PBS containing CD4,CD8, CD25,CD28, CD56, CD16, CD3 conjugated colored antibodies.

Total NK cell will be defined as the sum of CD56+CD16+CD3-, CD56-CD16+CD3- and CD56+CD16-CD3-. The total number of NK cells in the blood will be calculated by multiplying the % cells expressing the markers above and the total lymphocyte count in the blood.

4.2.8.3 Lymphoproliferative response

Peripheral blood will be drawn at baseline and within 2 weeks after HBOT completion. 6X 10^5 PBMC will be suspended in 200 microliter RPMI 1640/10% FCS and incubated with either medium alone, 20microgram/ml PHA, PWM diluted 1:2 X10^3 to induce suboptimal lymphocyte proliferative responses or 20 U/ml IL-2. PBMC will also be incubated for 7 days with medium or with ethanol inactivated candida antigen ttrated to the lowest concentration inducing an optimal lymphocyte proliferative response. During the lat 24 h cell will be exposed to 3H-thymidine. 3H-thymidine incorporation will be measured by a direct beta-counter.

PBMCs will be ashed twice in PBS with 3% FCS. 1X10^5 cells will be resuspended in 100 microliter PBS contatining CD4,CD8, CD62, CD28, CD45, CD3conjugated colored antibodies

4.2.8.4 NK cytotoxicity

NK cell cytotoxicity will be measured using K562 in c 51Cr release assay. PBMC (E) will be added in dif ferent concentrations to target cells (T). E/T cel ratios of 50/1, 25/1, 12.5/1 will be used. Each ratio will be incubated for 4 hours at 37 celsius. The plates will be centrifuged 10 mins, and 100microliter of supernatant will be transferred to new tubes and radioactivity will be determined. The spontateous release will be measure by incubating target cells with medium alone Maximum release will be measured by incubating target cells with 10% Triton X-100. The percentage of 51Cr release will be derermined by %Lysis = (sample – spontaneous) cpm / (maximum – spontaneous) cpm. The cytotoxicity per NK cell will be calculated as %Lysis / (%NK cells X number of effector cells total). Index of cytotoxicity in the blood will be calculated as number of NK cells in the blood X cytotoxicity per NK cell.

4.2.9 Microbiome

The Human Microbiome is the collection of all the microorganisms living in association with the human body. These communities consist of a variety of microorganisms including eukaryotes, archaea, bacteria and viruses. Bacteria in an average human body number ten times more than human cells, for a total of about 1000 more genes than are present in the human genome. However, because of their small size, microorganisms are only 1 to 3 percent of our body mass (that's 2 to 6 pounds of bacteria in a 200-pound adult). These microbes are essential for maintaining good health. For example, they produce some essential vitamins, extract nutrients from digested food, educate the immune systems to recognize dangerous invaders and even produce helpful anti-inflammatory compounds that might fight off other disease-causing microbes. (From the NIH Human Microbiome Project website: About the HMP).

4.2.9.1 Microbiome and human behavior:

The gut has been shown to have an effect on human behavior, while a broad spectrum antibiotic drug, Minocycline, has been recently suggested as a candidate treatment for depression [60] and schizophrenia [61]. Furthermore, an association between gastroenterological disease such as IBD and psychiatric morbidity (e.g. depression and anxiety) is well known, although precise mechanisms have not yet been shown.

Additionally, in a human study testing effects of probiotics on brain function, women receiving the probiotics had altered brain activity in regions associated with central processing of emotion and sensation [62]. Moreover, a pilot study conducted on 39 patients suffering from chronic fatigue syndrome demonstrated a significant decrease in anxiety symptoms among patients treated with *Lactobacillus casei* strain Shirota for two months, compared with placebo [63].

4.2.9.2 Gut-brain signaling:

It has long been known that the brain has a strong effect on the gut. The gastrointestinal tract is sensitive to emotion in a way that anger, anxiety, sadness, and elation can all trigger gut symptoms. However, it has only recently been shown that the cross-talk between these organs is bilateral and the gut has effects on the brain as well. There appear to be different levels of signaling from the gut to the brain. One is neuronal - by bi-directional signaling, between the enteric nervous system (ENS) and the CNS via the vagus nerve. The ENS is composed of approximately 100 million nerve cells, leading to be called "our second brain"[64]. Additional indirect effects of the gut on the brain include altering function of the endocrine system. Microbes are capable of producing neurotransmitters such as norepinephrine, serotonin, and dopamine, which have effects on the CNS. Additionally, the microbiome can affect host hormone levels. Finally, it has been shown that the microbiome can affect immune system components, altering cytokine levels and levels of inflammation.

4.2.9.3 Microbiome evaluation method:

Advances in bacterial genome sequencing is used for characterization of the human commensal bacterial community (microbiota) and its corresponding genome (microbiome). Using our cutting-edge facilities, 16S ribosomal RNA (rRNA) next-generation sequencing of fecal samples will be performed to identify bacteria present in the gut. 16S rRNA gene sequencing is a well-established method for studying phylogeny and taxonomy (the description, identification and evolutionary classification) of samples from complex microbial environments that are difficult to study. The test detects beneficial as well as pathogenic microorganisms associated with

specific infections, lifestyle choices, and gut conditions. To complete the picture of the gut ecology, the metatranscriptome (the complete mapping of messenger RNAs encoded by the organisms in a complex sample) will allow us to evaluate not only the identity of the specific organisms residing in the gut, but also their function.

5. Administration and regulation

5.1 Informed Consent

The investigator will obtain written informed consent from the parents of the patient participating in this study after adequate explanation of the aims, methods, objectives, and potential hazards of the study and prior to undertaking any study-related procedures. The investigator must utilize a consent form for documenting written informed consent. Informed consent will be appropriately signed and dated by the patient or the subject's legally authorized representative and the person obtaining consent.

5.2 Confidentiality

Subjects' anonymity will be strictly maintained and their identities will remain protected from unauthorized parties. The information is not to be disclosed to any third party (except for medical stuff or employees or agents directly involved in the conduct of the study or as required by law).

5.3 Study Files

The medical records will be maintained adequately to enable good data storage and latter on management. Subjects' clinical source documents will include (but not limited to) the following: subject's hospital/clinic/ hyperbaric unit records, physician's and nurse's notes, appointment book, original laboratory reports, X-ray, MRIs, and special assessment reports, consultant letters, screening and enrollment log, etc.

5.4 Randomization and handling of the control group

Since the diversity of the patients included in the study, after signing the informed consent is expected to be high, no stratifies criteria will be used. Patients will be randomized in 1:1 fashion to either HBOT or pharmaceutical group.

5.5 Adverse Events

An adverse event (AE) is any untoward medical occurrence in a clinical investigation subject administered a medicinal product and which does not necessarily have a causal relationship with this treatment. An AE can therefore be any unfavorable and unintended sign, symptom, or disease temporally associated with the use of a medicinal product, whether or not considered related to the medicinal product. Pre-existing events, which increase in severity or change in nature during or as a consequence of use of a medicinal product in human clinical trials, will also be considered AEs.

Any medical condition or clinically significant laboratory abnormality with an onset date before the screening visit and not related to study procedures is considered to be pre-existing, and should be documented in the case report form.

6. Statistical Considerations

6.1 Analysis Sets

6.1.1 Safety Analysis Set

The safety analysis set will consist of all subjects with whom the study treatment was initiated.

6.1.2 Primary Efficacy Analysis Set

The primary endpoint parameter: *improvement in fibromyalgia questioneers scores* will be measured following the 1st treatment period and separately after the crossed treatment period.
The primary efficacy analysis evaluation will include all subjects who completed the treatment period, had no major protocol violations.

6.1.2.1 Sample Size Considerations

According to Initiative on Methods, Measurement, and Pain Assessment in Clinical Trials (IMMPACT) recommendations, pain intensity reductions of 30% to 50% or more are considered useful. Therefore, the estimated sample size was calculated based on a reduction of 50% in the HBOT group and a reduction of 20% in the medication group, with a standard deviation of 40% [65, 66]. Assuming a power of 80%, and 5% two-sided level of significance, a total of 58 participants would be required, 29 participants in each arm. Considering a dropout rate of 15% the total sample size required is 70.

7. References

1. Schmidt-Wilcke, T. and D.J. Clauw, *Fibromyalgia: from pathophysiology to therapy.* Nature Reviews Rheumatology, 2011. **7**(9): p. 518-527.
2. Buskila, D., *Developments in the scientific and clinical understanding of fibromyalgia.* Arthritis research & therapy, 2009. **11**(5): p. 242.
3. Yunus, M.B. *Fibromyalgia and overlapping disorders: the unifying concept of central sensitivity syndromes*. in *Seminars in arthritis and rheumatism*. 2007. Elsevier.
4. Yunus, M.B., *Role of central sensitization in symptoms beyond muscle pain, and the evaluation of a patient with widespread pain.* Best Practice & Research Clinical Rheumatology, 2007. **21**(3): p. 481-497.
5. Staud, R., et al., *Diffuse noxious inhibitory controls (DNIC) attenuate temporal summation of second pain in normal males but not in normal females or fibromyalgia patients.* Pain, 2003. **101**(1): p. 167-174.
6. Gracely, R.H., et al., *Functional magnetic resonance imaging evidence of augmented pain processing in fibromyalgia.* Arthritis & Rheumatism, 2002. **46**(5): p. 1333-1343.
7. Napadow, V., et al., *Intrinsic brain connectivity in fibromyalgia is associated with chronic pain intensity.* Arthritis & Rheumatism, 2010. **62**(8): p. 2545-2555.
8. Napadow, V., et al., *Brief report: decreased intrinsic brain connectivity is associated with reduced clinical pain in fibromyalgia.* Arthritis & Rheumatism, 2012. **64**(7): p. 2398-2403.
9. Hampson, J., et al., *Pregabalin alters default mode network activity in fibromyalgia.* The Journal of Pain, 2012. **13**(4): p. S30.
10. Hargrove, J.B., et al. *Symptom Improvement in Fibromyalgia Patients Is Related to Reduced Network Connectivity As Measured by EEG Coherence*. in *ARTHRITIS AND RHEUMATISM*. 2012. WILEY-BLACKWELL 111 RIVER ST, HOBOKEN 07030-5774, NJ USA.
11. Hadanny, A. and S. Efrati, *Treatment of persistent post-concussion syndrome due to mild traumatic brain injury: current status and future directions.* Expert review of neurotherapeutics, 2016. **16**(8): p. 875-87.
12. Tal, S., et al., *Hyperbaric oxygen may induce angiogenesis in patients suffering from prolonged post-concussion syndrome due to traumatic brain injury.* Restorative neurology and neuroscience, 2015. **33**(6): p. 943-51.
13. Hadanny, A., et al., *Hyperbaric oxygen can induce neuroplasticity and improve cognitive functions of patients suffering from anoxic brain damage.* Restorative neurology and neuroscience, 2015. **33**(4): p. 471-86.
14. Boussi-Gross, R., et al., *Improvement of memory impairments in poststroke patients by hyperbaric oxygen therapy.* Neuropsychology, 2015. **29**(4): p. 610-21.
15. Efrati, S. and E. Ben-Jacob, *Reflections on the neurotherapeutic effects of hyperbaric oxygen.* Expert review of neurotherapeutics, 2014. **14**(3): p. 233-6.
16. Boussi-Gross, R., et al., *Hyperbaric oxygen therapy can improve post concussion syndrome years after mild traumatic brain injury - randomized prospective trial.* PLoS One, 2013. **8**(11): p. e79995.
17. Efrati, S., et al., *Hyperbaric oxygen induces late neuroplasticity in post stroke patients--randomized, prospective trial.* PLoS One, 2013. **8**(1): p. e53716.

18. Mu, J., et al., *Delayed hyperbaric oxygen therapy induces cell proliferation through stabilization of cAMP responsive element binding protein in the rat model of MCAo-induced ischemic brain injury.* Neurobiol Dis, 2013. **51**: p. 133-43.
19. Yang, Y.J., et al., *Hyperbaric oxygen induces endogenous neural stem cells to proliferate and differentiate in hypoxic-ischemic brain damage in neonatal rats.* Undersea Hyperb Med, 2008. **35**(2): p. 113-29.
20. Chang, C.C., et al., *Damage of white matter tract correlated with neuropsychological deficits in carbon monoxide intoxication after hyperbaric oxygen therapy.* J Neurotrauma, 2009. **26**(8): p. 1263-70.
21. Vilela, D.S., P.R. Lazarini, and C.F. Da Silva, *Effects of hyperbaric oxygen therapy on facial nerve regeneration.* Acta Otolaryngol, 2008. **128**(9): p. 1048-52.
22. Haapaniemi, T., et al., *Hyperbaric oxygen treatment enhances regeneration of the rat sciatic nerve.* Exp Neurol, 1998. **149**(2): p. 433-8.
23. Bradshaw, P.O., et al., *Effect of hyperbaric oxygenation on peripheral nerve regeneration in adult male rabbits.* Undersea Hyperb Med, 1996. **23**(2): p. 107-13.
24. Mukoyama, M., M. Iida, and I. Sobue, *Hyperbaric oxygen therapy for peripheral nerve damage induced in rabbits with clioquinol.* Exp Neurol, 1975. **47**(3): p. 371-80.
25. Efrati, S., et al., *Hyperbaric oxygen therapy can diminish fibromyalgia syndrome--prospective clinical trial.* PLoS One, 2015. **10**(5): p. e0127012.
26. Yildiz, S., et al., *A new treatment modality for fibromyalgia syndrome: hyperbaric oxygen therapy.* The Journal of international medical research, 2004. **32**(3): p. 263-7.
27. Hadanny, A. and S. Efrati, *Treatment of persistent post-concussion syndrome due to mild traumatic brain injury: current status and future directions.* Expert review of neurotherapeutics, 2016. **16**(8): p. 875-887.
28. Malec, J.F., et al., *The Mayo classification system for traumatic brain injury severity.* Journal of neurotrauma, 2007. **24**(9): p. 1417-1424.
29. Tal, S., et al., *Hyperbaric oxygen may induce angiogenesis in patients suffering from prolonged post-concussion syndrome due to traumatic brain injury.* Restorative neurology and neuroscience, 2015. **33**(6): p. 943-951.
30. Figueroa, X.A. and J.K. Wright, *Hyperbaric oxygen B-level evidence in mild traumatic brain injury clinical trials.* Neurology, 2016. **87**(13): p. 1400-1406.
31. Günther, A., et al., *Reduced infarct volume and differential effects on glial cell activation after hyperbaric oxygen treatment in rat permanent focal cerebral ischaemia.* European Journal of Neuroscience, 2005. **21**(11): p. 3189-3194.
32. Milligan, E.D. and L.R. Watkins, *Pathological and protective roles of glia in chronic pain.* Nature reviews neuroscience, 2009. **10**(1): p. 23-36.
33. Smith, H.S., *Activated microglia in nociception.* Pain Physician, 2010. **13**(3): p. 295-304.
34. Calvert, J.W., J. Cahill, and J.H. Zhang, *Hyperbaric oxygen and cerebral physiology.* Neurological research, 2007. **29**(2): p. 132-141.
35. Niklas, A., et al., *Continuous measurements of cerebral tissue oxygen pressure during hyperbaric oxygenation—HBO effects on brain edema and necrosis after severe brain trauma in rabbits.* Journal of the Neurological Sciences, 2004. **219**(1): p. 77-82.
36. Ablin, J.N. and D. Buskila, *Update on the genetics of the fibromyalgia syndrome.* Best Practice & Research Clinical Rheumatology, 2015. **29**(1): p. 20-28.
37. Al-Allaf, A., et al., *A case–control study examining the role of physical trauma in the onset of fibromyalgia syndrome.* Rheumatology, 2002. **41**(4): p. 450-453.

38. Buskila, D., et al., *Increased rates of fibromyalgia following cervical spine injury. A controlled study of 161 cases of traumatic injury.* Arthritis & Rheumatism, 1997. **40**(3): p. 446-452.
39. Buskila, D., F. Atzeni, and P. Sarzi-Puttini, *Etiology of fibromyalgia: the possible role of infection and vaccination.* Autoimmunity reviews, 2008. **8**(1): p. 41-43.
40. Wolfe, F., et al. *2016 Revisions to the 2010/2011 fibromyalgia diagnostic criteria*. in *Seminars in Arthritis and Rheumatism*. 2016. Elsevier.
41. Ablin, J., et al., *Guidelines for the diagnosis and treatment of the fibromyalgia syndrome.* Harefuah, 2013. **152**(12): p. 742-7, 751, 750.
42. Hawker, G.A., et al., *Measures of adult pain: Visual analog scale for pain (vas pain), numeric rating scale for pain (nrs pain), mcgill pain questionnaire (mpq), short-form mcgill pain questionnaire (sf-mpq), chronic pain grade scale (cpgs), short form-36 bodily pain scale (sf-36 bps), and measure of intermittent and constant osteoarthritis pain (icoap).* Arthritis care & research, 2011. **63**(S11): p. S240-S252.
43. Tory McJunkin, M., *Reliability and validity of the Global Pain Scale with chronic pain sufferers.* Pain physician, 2011. **14**: p. 61-70.
44. Ferguson, L. and J. Scheman, *Patient global impression of change scores within the context of a chronic pain rehabilitation program.* The Journal of Pain, 2009. **10**(4): p. S73.
45. Buskila, D. and L. Neumann, *Assessing functional disability and health status of women with fibromyalgia: validation of a Hebrew version of the Fibromyalgia Impact Questionnaire.* The Journal of rheumatology, 1996. **23**(5): p. 903-906.
46. Wolfe, F. and W. Häuser, *Fibromyalgia diagnosis and diagnostic criteria.* Annals of medicine, 2011. **43**(7): p. 495-502.
47. Neumann, L., A. Berzak, and D. Buskila. *Measuring health status in Israeli patients with fibromyalgiasyndrome and widespread pain and healthy individuals: Utility of the Short Form 36-item health survey (SF-36)*. in *Seminars in arthritis and rheumatism*. 2000. Elsevier.
48. Williams, D.A. and L.M. Arnold, *Measures of fibromyalgia: Fibromyalgia Impact Questionnaire (FIQ), Brief Pain Inventory (BPI), Multidimensional Fatigue Inventory (MFI-20), Medical Outcomes Study (MOS) Sleep Scale, and Multiple Ability Self-Report Questionnaire (MASQ).* Arthritis care & research, 2011. **63**(S11): p. S86-S97.
49. Beck, A.T., R.A. Steer, and G.K. Brown, *Beck depression inventory.* 1996.
50. Doniger, G.M. *Mindstreams Computerized Cognitive Tests: Test Descriptions. Available: http://www.mirror.upsite.co.il/uploaded/files/1383_e7d7d3d98c924f036d3123733419149d.pdf. Accessed 05 July 2013*. 2007 [cited 2013 05 July]; Available from: http://www.mirror.upsite.co.il/uploaded/files/1383_e7d7d3d98c924f036d3123733419149d.pdf.
51. Doniger, G.M. *Guide to MindStreams Normative Data. Available: http://www.mirror.upsite.co.il/uploaded/files/1383_b44d4786c91058be301cb09a94ba70f4.pdf. Accessed 05 July 2013*. 2012; Available from: http://www.mirror.upsite.co.il/uploaded/files/1383_b44d4786c91058be301cb09a94ba70f4.pdf.
52. Zygouris, S. and M. Tsolaki, *Computerized cognitive testing for older adults: a review.* Am J Alzheimers Dis Other Demen, 2015. **30**(1): p. 13-28.

53. Klekociuk, S.Z., et al., *Reducing false positive diagnoses in mild cognitive impairment: the importance of comprehensive neuropsychological assessment.* Eur J Neurol, 2014. **21**(10): p. 1330-6, e82-3.
54. Saunders, N.L. and M.J. Summers, *Attention and working memory deficits in mild cognitive impairment.* J Clin Exp Neuropsychol, 2010. **32**(4): p. 350-7.
55. Bird, C.M., et al., *Monitoring cognitive changes: psychometric properties of six cognitive tests.* Br J Clin Psychol, 2004. **43**(Pt 2): p. 197-210.
56. Castillo-Saavedra, L., et al., *Clinically Effective Treatment of Fibromyalgia Pain With High-Definition Transcranial Direct Current Stimulation: Phase II Open-Label Dose Optimization.* The journal of pain : official journal of the American Pain Society, 2016. **17**(1): p. 14-26.
57. Reches, A., et al., *A novel electroencephalography-based tool for objective assessment of network dynamics activated by nociceptive stimuli.* European journal of pain, 2016. **20**(2): p. 250-62.
58. Reches, A., et al., *Preliminary investigation of Brain Network Activation (BNA) and its clinical utility in sport-related concussion.* Brain injury, 2017: p. 1-10.
59. Kiefer, A.W., et al., *Brain Network Activation as a Novel Biomarker for the Return-to-Play Pathway Following Sport-Related Brain Injury.* Frontiers in neurology, 2015. **6**: p. 243.
60. Soczynska, J.K., et al., *Novel therapeutic targets in depression: minocycline as a candidate treatment.* Behavioural brain research, 2012. **235**(2): p. 302-317.
61. Miyaoka, T., *Clinical potential of minocycline for schizophrenia.* CNS & Neurological Disorders-Drug Targets (Formerly Current Drug Targets-CNS & Neurological Disorders), 2008. **7**(4): p. 376-381.
62. Tillisch, K., et al., *Consumption of fermented milk product with probiotic modulates brain activity.* Gastroenterology, 2013. **144**(7): p. 1394-1401. e4.
63. Rao, A.V., et al., *A randomized, double-blind, placebo-controlled pilot study of a probiotic in emotional symptoms of chronic fatigue syndrome.* Gut Pathogens, 2009. **1**(1): p. 6.
64. Gershon, M.D., *The enteric nervous system: a second brain.* Hospital Practice, 1999. **34**(7): p. 31-52.
65. Moore, R.A., et al., *Systematic review of enriched enrolment, randomised withdrawal trial designs in chronic pain: a new framework for design and reporting.* Pain, 2015. **156**(8): p. 1382-1395.
66. Dworkin, R.H., et al., *Interpreting the clinical importance of treatment outcomes in chronic pain clinical trials: IMMPACT recommendations.* The journal of pain, 2008. **9**(2): p. 105-121.
